# Fatigue Performance and Pore Characteristics of SBS/Micro Carbon Fiber Composite-Modified Asphalt Concrete for Ultra-Thin Overlays

**DOI:** 10.3390/polym18172062

**Published:** 2026-08-25

**Authors:** Xiaodong Yang, Mingxin Liu, Xiaojin Lu, Jingyu Xiao, Jifa Liu, Quanman Zhao

**Affiliations:** 1 School of Transportation, Kashi University, Kashi 844000, China; 2 School of Transportation Engineering, Shandong Jianzhu University, Jinan 250101, China; 3 School of Transportation, Southeast University, Nanjing 211189, China; 4 Shandong Taishan Communications Planning and Design Consulting Co., Ltd., Tai’an 271000, China

**Keywords:** styrene–butadiene–styrene/micro carbon fiber composite-modified asphalt, ultra-thin overlay, freeze–thaw cycling, water immersion, fatigue performance, pore structure, X-ray computed tomography, adaptive-network-based fuzzy inference system

## Abstract

Durability deterioration and interlayer bonding failure of ultra-thin overlays remain critical challenges under coupled environmental and mechanical actions. Although environmental damage to asphalt mixtures has been widely investigated, the relationship between pore-structure evolution and interlayer fatigue deterioration in polymer-composite-modified ultra-thin overlays incorporating styrene–butadiene–styrene (SBS) and micro carbon fiber (MCF) remains insufficiently understood. This study therefore extends existing research by clarifying this relationship under freeze–thaw cycling and water immersion. Three-point bending and direct shear fatigue tests were conducted to evaluate bending and interlayer shear fatigue performance, respectively, while nanoindentation and X-ray computed tomography (CT) were used to characterize micromechanical properties and three-dimensional pore-structure evolution. The results showed that five freeze–thaw cycles reduced the bending fatigue life by 75.3% and the interlayer shear fatigue life by 49.6%, while six days of water immersion reduced the interlayer shear fatigue life by 55.1%. Freeze–thaw cycling promoted open-pore and pore-throat development and increased total porosity by 23.9%, contributing to aggregate displacement and redistribution of the internal skeleton. In contrast, immersion increased the proportion of small and closed pores, while isolated pores concentrated near the interlayer weakened interlayer shear resistance. Although immersion caused greater reductions in hardness and modulus, freeze–thaw-induced pore development was associated with greater deterioration in bending fatigue performance. Furthermore, an adaptive-network-based fuzzy inference system (ANFIS) was developed to predict pore tortuosity from equivalent diameter, shape factor, and porosity, with testing errors ranging from 0.102 to 0.129 for untreated, freeze–thaw, and immersed specimens. An exponential relationship was further identified between tortuosity and the pore comprehensive effect index (PCEI), providing a quantitative approach for characterizing pore connectivity and evaluating environmental deterioration in polymer-composite-modified asphalt concrete.

## 1. Introduction

With the continuous development of transportation networks and the increase in traffic volume, the design and performance evaluation of asphalt pavements have become increasingly important. During service, asphalt pavements are exposed not only to repeated vehicle loads but also to various environmental actions [1]. Among these factors, water infiltration and freeze–thaw cycling can significantly alter the pore structure and mechanical performance of asphalt mixtures [2], thereby affecting pavement durability and service reliability. Therefore, evaluating the performance deterioration of asphalt mixtures under water immersion and freeze–thaw conditions is essential for understanding their long-term service behavior [3,4,5].

Polymer modification has been widely used to improve the performance of asphalt materials. Styrene–butadiene–styrene (SBS) is one of the most commonly used polymer modifiers and can improve the elasticity, deformation resistance, and fatigue performance of asphalt binders [6,7,8]. Because ultra-thin overlays have a relatively small structural thickness, the performance of the asphalt mixture and the bonding condition between layers have an important influence on pavement durability [9]. Zhao et al. [10] studied micro carbon fiber (MCF) composite-modified high-viscosity asphalt and found that the incorporation of MCF improved its high-temperature performance and mechanical properties. Zhao et al. [11] further investigated SBS/MCF-modified asphalt mixtures for ultra-thin overlays and showed that the composite-modified mixture exhibited favorable road performance. However, the fatigue deterioration and pore-structure evolution of SBS/MCF-modified ultra-thin overlays under freeze–thaw and immersion conditions still require further investigation.

Studies have shown that immersion and freeze–thaw cycling gradually weaken the adhesion between asphalt and aggregate [12,13] and alter the pore structure, leading to increased porosity, changes in pore connectivity, and nonuniform pore distribution [14]. Changes in pore structure can cause different degrees of damage to asphalt mixtures and reduce their mechanical properties, including compressive strength, shear strength, and fatigue life [15,16]. Furthermore, immersion and freeze–thaw cycling weaken the adhesion between aggregates and asphalt mastic, thereby significantly affecting the strength and durability of asphalt mixtures [17]. The environmental stability of polymer-modified asphalt also affects mixture durability. Xing et al. [18] investigated SBS-modified asphalt in a salt-solution environment and found that environmental exposure altered its rheological and aging characteristics. These findings indicate that both changes in the polymer-modified binder and changes in the internal structure of the mixture should be considered when evaluating environmental deterioration.

The internal pore structure of asphalt mixtures is a key factor affecting their macroscopic mechanical properties, especially fatigue life [19,20]. To precisely characterize this complex microstructure, X-ray computed tomography (CT) combined with digital image processing (DIP) has been widely adopted [21,22]. As a nondestructive testing technique, X-ray CT can reconstruct the three-dimensional pore structure of asphalt mixtures and reveal changes in pore number, pore size distribution, and pore connectivity [23]. For instance, Li et al. [19] used X-ray CT to investigate the pore distribution of porous asphalt concrete (PAC), finding that the equivalent diameters of connected pores were mainly distributed between 0.5 mm and 5 mm and establishing a relationship among porosity, connected porosity, and mineral aggregate composition. Ren et al. [24] analyzed asphalt concrete with different porosities using three-dimensional CT scanning, and found that the pore coordination number increased with increasing porosity. Alawneh et al. [25] used X-ray CT to characterize asphalt concrete mixtures after freeze–thaw cycling and observed clear changes in the internal air-void structure. Xu et al. [26] divided internal pores into isolated, semi-connected, connected, and open pores and found that different pore types exhibited different evolution and connectivity characteristics during freeze–thaw cycling. These results indicate that freeze–thaw damage is closely associated with changes in pore type and connectivity.

Based on the pore structures obtained from CT scans, the researchers have further investigated the relationship between pore characteristics and the macroscopic properties of the asphalt mixtures. Ling et al. [3] reconstructed a three-dimensional pore-network model of porous asphalt mixture (PAM) used in cast-in-place semi-flexible pavement (PFP) based on CT images. The permeability simulation results showed that connected porosity had a greater effect on fluid permeability than pore and throat sizes, and vertical permeability was higher than horizontal permeability, reflecting the anisotropic characteristics of the pore structure. Zhao et al. [27] analyzed the composite structure between new and recycled asphalt mixture layers using CT scanning. They found that, after freeze–thaw cycling, the pore volume and throat radius of the lower recycled mixture layer (AC-20) increased more markedly than those of the upper newly mixed mixture layer (SMA-13), indicating that the pore structure of recycled materials was more sensitive to moisture and that the interlayer region became a weak area in terms of water resistance [28]. Ren et al. [29] used CT scanning to evaluate the mesoscopic characteristics of fiber-reinforced porous asphalt under dynamic water action and further demonstrated the relationship between water-induced deterioration and pore-structure changes.

Under the combined action of loading and environmental factors, fatigue loading can further aggravate pore-structure deterioration and consequently accelerate the loss of the mixture performance. Zhang et al. [30] used low-field nuclear magnetic resonance (NMR) technology to investigate pore evolution in asphalt mixtures under cyclic loading and found that irreversible plastic deformation caused by dynamic water pressure promoted microcrack propagation and pore connection. Xiong et al. [12] used a self-developed water-flow-salt-erosion test device to simulate the sulfate-water-temperature-load coupling. They found that water erosion significantly accelerated sulfate damage and reduced splitting strength. The deterioration mechanism was related to weakened asphalt–aggregate adhesion and salt crystalline erosion [31]. The interlayer bonding condition also has an important influence on the fatigue performance of overlay structures. Jin et al. [32] investigated the bonding performance between asphalt concrete overlays and Portland cement concrete using inclined shear fatigue tests and found that the interlayer condition significantly affected fatigue behavior. The structural design and interlayer behavior of ultra-thin overlays are therefore important factors affecting their service performance [9,33].

Among environmental actions, freeze–thaw cycling has a particularly significant impact on the internal pore structure of asphalt mixtures [34,35]. Xu et al. [36] used X-ray CT to investigate internal structural evolution during freeze–thaw cycling and found that pores were mainly characterized by the expansion of existing pores, connection of isolated pores, and formation of new pores. Wu et al. [37] combined X-ray CT and mercury intrusion porosimetry (MIP) to investigate the effect of the snow-melting agent Mafilon on the microscopic pore structure of anti-freezing asphalt pavement and found that freeze–thaw cycling mainly increased the number of medium-sized pores (0.2–0.6 mm), and that freeze–thaw action and pore-structure evolution promoted each other, leading to more severe material deterioration. Xiao et al. [4] used X-ray CT to analyze an asphalt-mixture waterproofing layer for high-speed railways after freeze–thaw cycling. By dividing the specimens into core, middle shell and outer layer, they identified a “lag effect” in freeze–thaw damage, in which deterioration gradually developed from the surface toward the interior, accompanied by the formation and propagation of microcracks. Guo et al. [38] combined the semi-circular bending fatigue test (SCB) with the digital speckle correlation method (DSCM) to investigate the fatigue damage of composite crumb-rubber-modified asphalt mixtures and found that an increase in the number of freeze–thaw cycles led to an increase in the peak value of stiffness modulus attenuation (PV) and a reduction in horizontal strain density (DE), and they subsequently established a fatigue damage model with good correlation.

Based on the above analysis, although considerable research has been conducted on the effects of freeze–thaw cycling and water immersion on asphalt mixtures, several issues related to ultra-thin overlays still require further investigation. First, most studies have focused on conventional asphalt mixtures or single-layer structures, whereas the fatigue behavior of SBS/MCF composite-modified asphalt concrete with an interlayer has received relatively limited attention under freeze–thaw and immersion conditions. Second, although CT technology has been widely used to characterize the pore structure of asphalt mixtures, quantitative comparisons of pore evolution among different layers, particularly in the interlayer region of ultra-thin overlay specimens, remain insufficient. Third, the relationship between pore characteristics and fatigue deterioration still needs to be further established for polymer-composite-modified ultra-thin overlays. These limitations affect both the understanding of environmental damage mechanisms and the evaluation of the long-term performance of ultra-thin overlays. To address these issues, this study combines fatigue behavior, interlayer micromechanical properties, three-dimensional pore evolution, and pore-tortuosity prediction to investigate the environmental deterioration of SBS/MCF composite-modified ultra-thin overlays. Specifically, this study investigates SBS/MCF composite-modified asphalt concrete for ultra-thin overlays under freeze–thaw cycling and water immersion. Three-point bending and direct shear fatigue tests are used to evaluate bending and interlayer shear fatigue performance, respectively, while nanoindentation is used to analyze changes in micromechanical properties. X-ray CT is used to reconstruct the three-dimensional pore structure and analyze pore volume, pore type, pore-throat characteristics, and pore distribution near the interlayer. Finally, considering the increasing application of data-driven methods in asphalt performance prediction and optimization [39,40], an adaptive-network-based fuzzy inference system (ANFIS) is used to predict pore tortuosity from pore characteristics, and the relationship between tortuosity and the pore comprehensive effect index (PCEI) is further analyzed. This study provides a basis for understanding the fatigue deterioration and pore evolution of polymer-composite-modified ultra-thin overlays under freeze–thaw cycling and water immersion.

## 2. Materials and Methods

### 2.1. Materials

**Figure 1:**
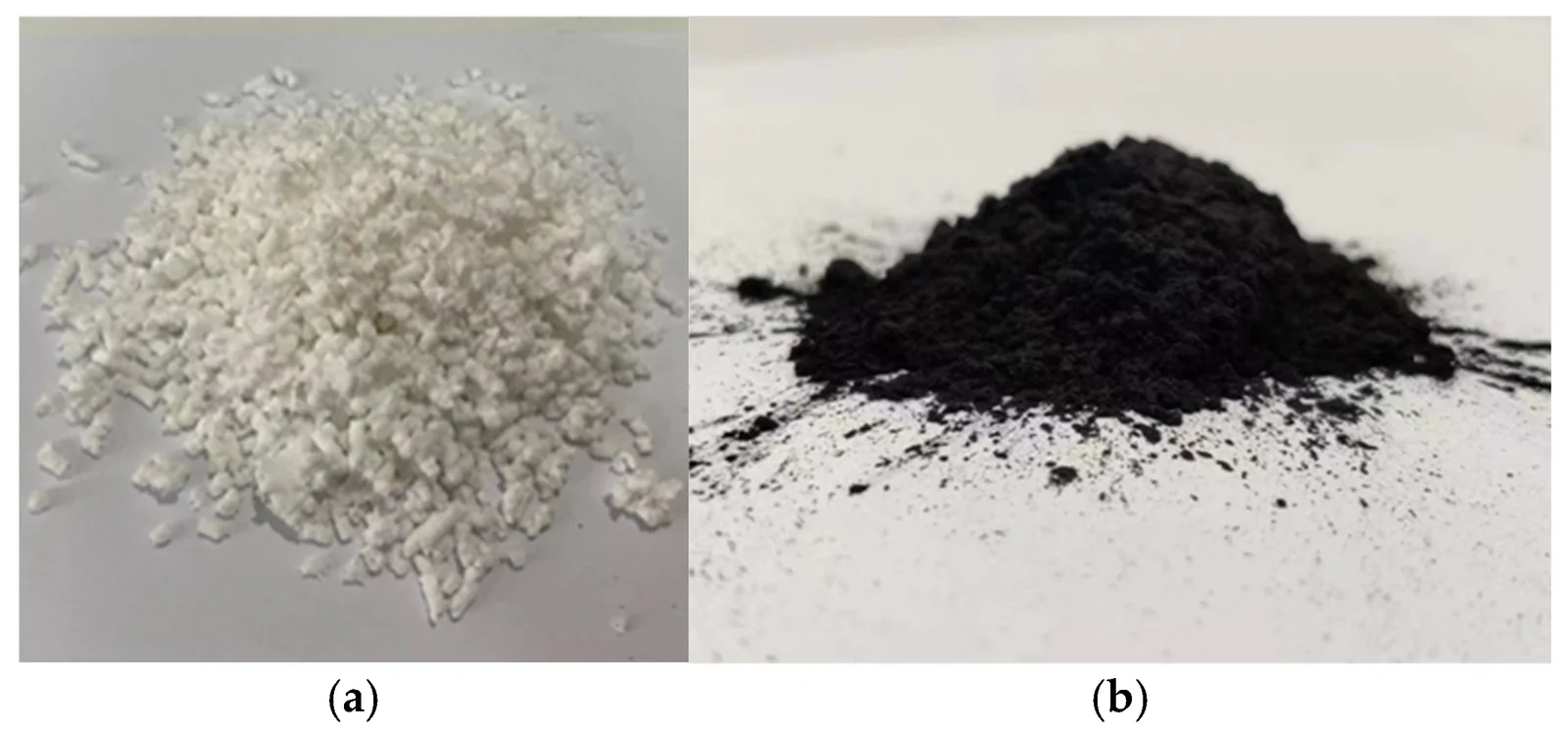
Representative photographs of the main polymer-modification materials: (**a**) SBS modifier; (**b**) micro carbon fiber (MCF).

**Figure 2:**
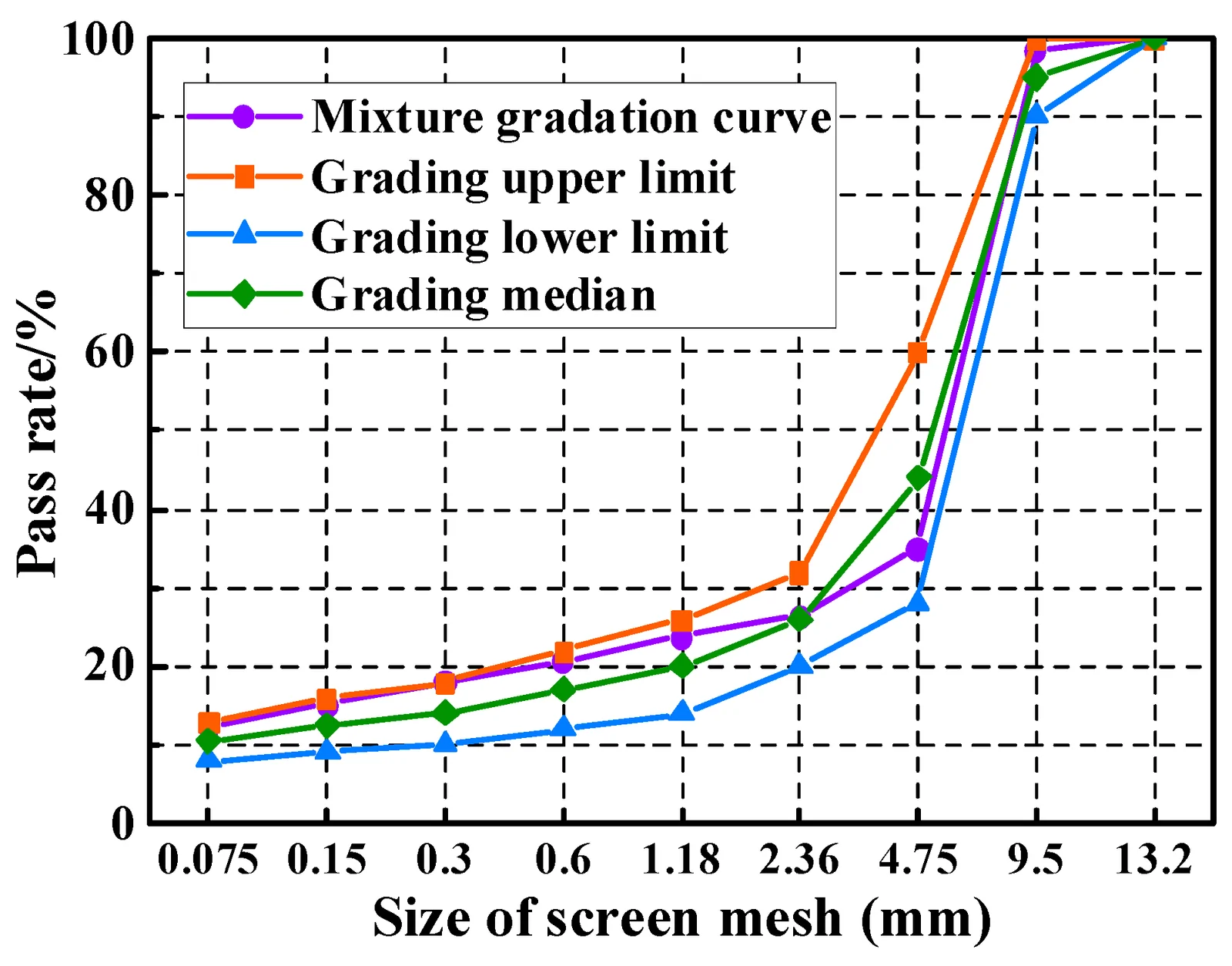
Mixture gradation design curve.

The test specimens used in the experiment were composed of the upper and lower layers of SMA-10 asphalt mixture to simulate the original pavement and the ultra-thin overlay structure. All asphalt binders used in the mixtures were SBS/MCF composite-modified asphalt, and the corresponding technical properties are presented in Table 1. The SBS/MCF composite-modified asphalt was prepared using 6% SBS and 0.8% MCF by mass of the base asphalt. The MCF used in this study had a specification of 1500 mesh, a density of 1.8 g/cm^3^, a tensile strength of 4900 MPa, and an elastic modulus of 230 GPa. Representative photographs of the SBS modifier and MCF used in this study are shown in Figure 1. The aggregates used were basalt crushed stones with particle sizes of 5–10 mm and manufactured sand with particle sizes of 0–3 mm, and the filler was limestone powder. The key passing percentages of the designed SMA-10 gradation were 13.2 mm (100%), 9.5 mm (98.4%), 4.75 mm (36.0%), 2.36 mm (27.5%), and 0.075 mm (13.2%), and the gradation curve is shown in Figure 2 [41].

### 2.2. Methods

**Figure 3:**
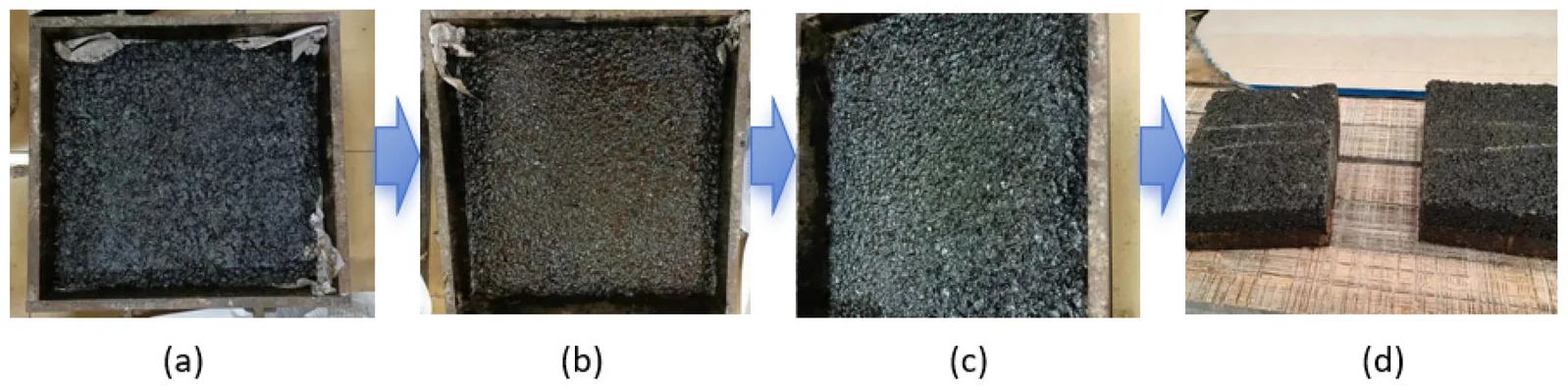
Preparation of rutting slabs: (**a**) single-layer rutting slab; (**b**) application of tack coat; (**c**) complete emulsion breaking; (**d**) double-layer rutting slab.

The double-layer rutting slabs were prepared by compacting the lower SMA-10 layer, applying the tack coat, allowing the emulsion to break completely, and then compacting the upper SMA-10 layer. The preparation process is shown in Figure 3.

Water immersion and freeze–thaw cycles represent important environmental actions for asphalt mixtures during service. Investigating their damage and deterioration mechanisms is essential for enhancing the long-term performance of asphalt mixtures. The specific freeze–thaw and immersion conditions adopted in this study were as follows: Based on the rapid freeze–thaw procedure, the freezing temperature was set at −18 °C and the thawing temperature at 60 °C. Each freeze–thaw cycle lasted 24 h. Prior to the first cycle, the specimens were immersed in water for 2 h to ensure complete water immersion. The number of freeze–thaw cycles applied in the test was 0, 1, 3, and 5.

For the immersion test, specimens were fully submerged in a constant-temperature water tank maintained at 25 °C. Since prolonged immersion leads to severe degradation of interlayer bonding performance and does not reflect real-world conditions, the immersion durations selected were 0, 2, 4, and 6 days. These conditioning levels were selected to characterize the progressive deterioration of fatigue performance while avoiding excessive damage to the interlayer structure.

**Figure 4:**
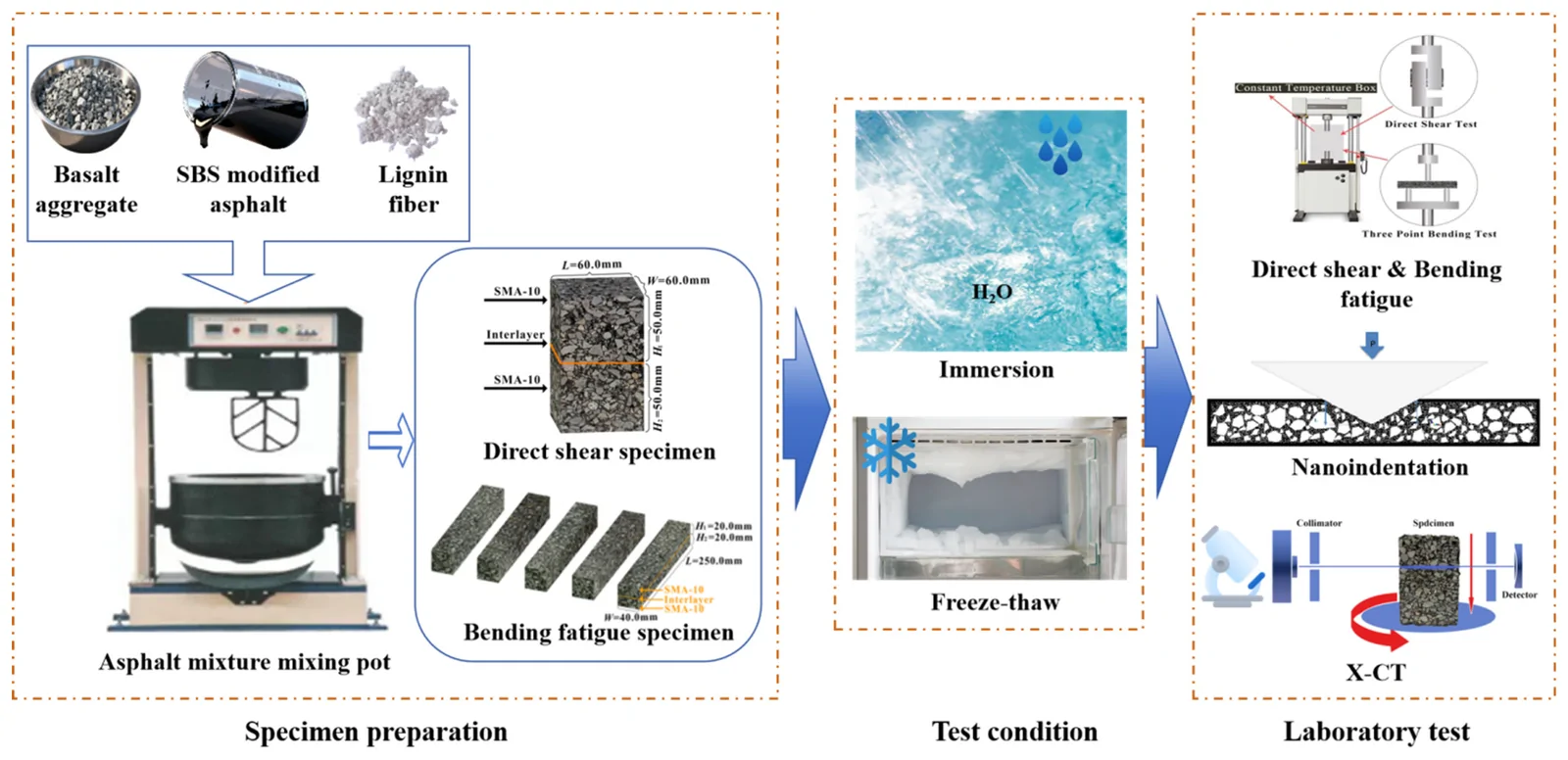
The test flowchart.

To elucidate the damage process of asphalt mixtures under both immersion and freeze–thaw conditions, specimens for direct shear and fatigue tests were first prepared according to the gradation curve shown in Figure 2, and then conditioned under the above protocols. To thoroughly understand the deterioration mechanisms, direct shear fatigue tests and three-point bending fatigue tests were employed to evaluate changes in macroscopic properties. Nanoindentation and CT scanning tests were further utilized to investigate the degradation of micromechanical properties and internal microstructure. The experimental flowchart is illustrated in Figure 4.

#### 2.2.1. Fatigue Tests of Asphalt Mixtures

**Figure 5:**
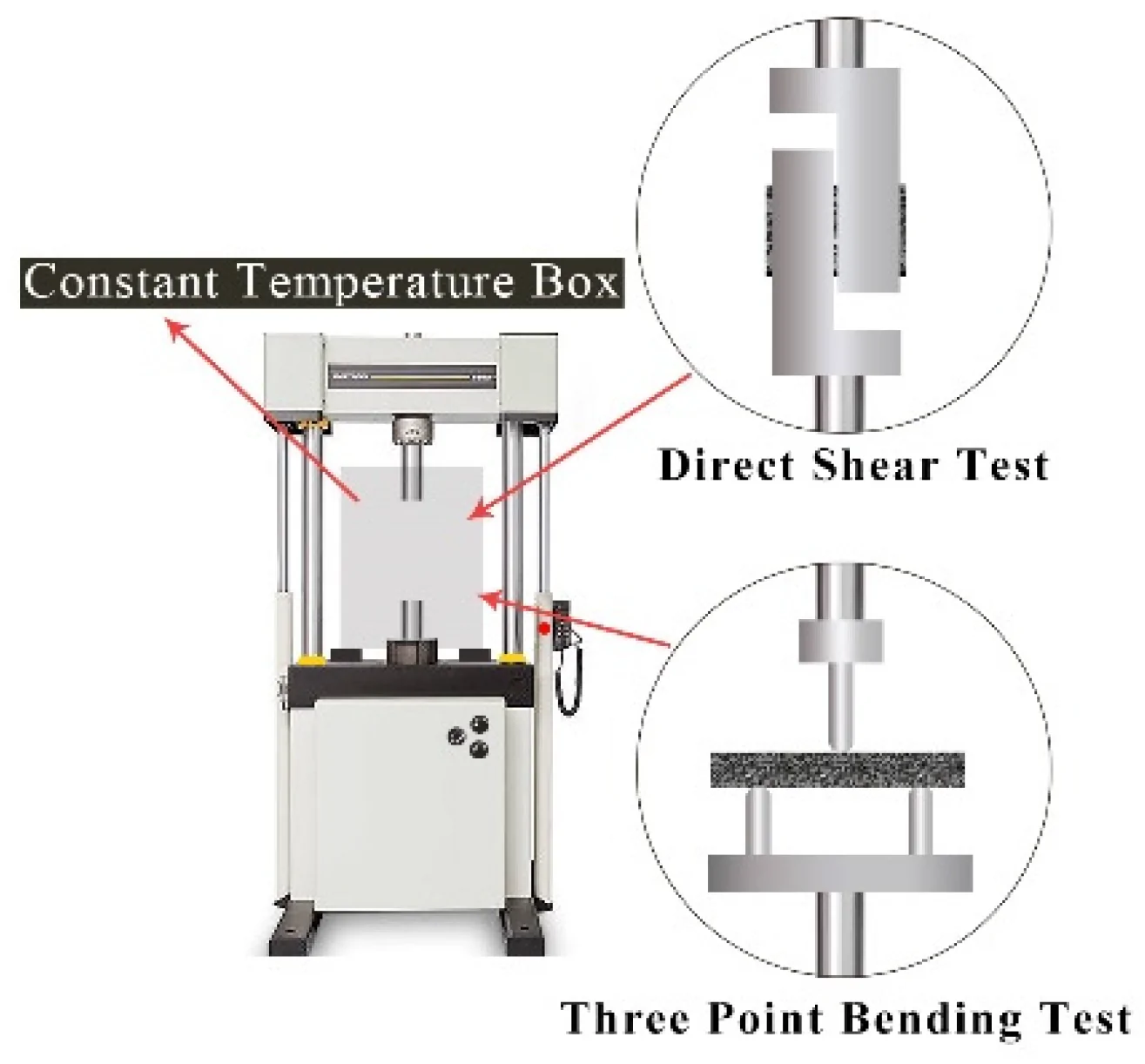
Indoor test equipment schematic diagram.

During service, asphalt pavement is subjected not only to vertical vehicle loads but also to shear stresses at the interlayer due to vehicle acceleration and braking. To evaluate the overall fatigue performance of the asphalt mixture and the fatigue performance at the interlayer, three-point bending fatigue tests and direct shear fatigue tests were conducted to evaluate bending fatigue performance and interlayer shear fatigue performance, respectively. The fatigue tests were conducted at 25 °C, with a stress ratio of 0.3 and a loading frequency of 10 Hz. Fatigue life was defined as the number of loading cycles at specimen failure. The test equipment and test mold are exhibited in Figure 5.

#### 2.2.2. Nanoindentation Test

**Figure 6:**
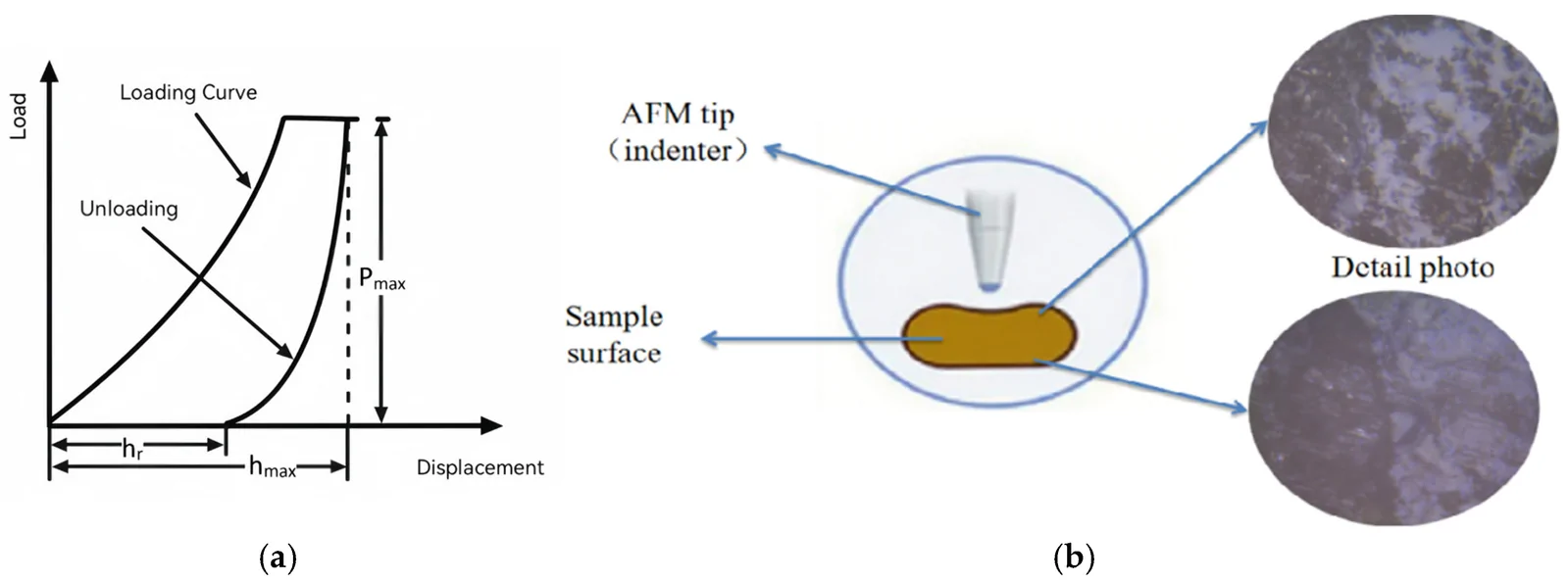
Nanoindentation test: (**a**) schematic diagram of the loading curve; (**b**) schematic diagram of the experimental setup.

Nanoindentation is a micromechanical testing technique that involves probing a smooth sample surface with a diamond tip at the nanoscale. During the test, the load and displacement of the indenter were recorded as it penetrated the surface, resulting in a load-displacement curve during both loading and unloading phases, as shown in Figure 6a. By analyzing the resulting curve using appropriate computational models, material properties such as hardness, Young’s modulus, and viscoelastic parameters can be derived.

To evaluate the effects of immersion and freeze–thaw cycling on the micromechanical properties of asphalt mixtures, specimens measuring 15 mm × 15 mm × 5 mm were prepared and subjected to both immersion and freeze–thaw conditioning. Prior to testing, the surfaces of the specimens were progressively polished using sandpaper of 200, 400, 800, 1200, and 2000 grit to fully expose the asphalt mixture.

Studies have shown that the ITZ is highly susceptible to cracking under mechanical and environmental loading, making its characterization essential for understanding the crack resistance of asphalt mixtures. Therefore, in this study, a Berkovich indenter was used in load-controlled mode to perform nanoindentation tests within the ITZ. For each specimen, a total of 20 test points were selected for micromechanical characterization. A schematic diagram of the experimental setup is shown in Figure 6b.

#### 2.2.3. CT Scanning Test

**Figure 7:**
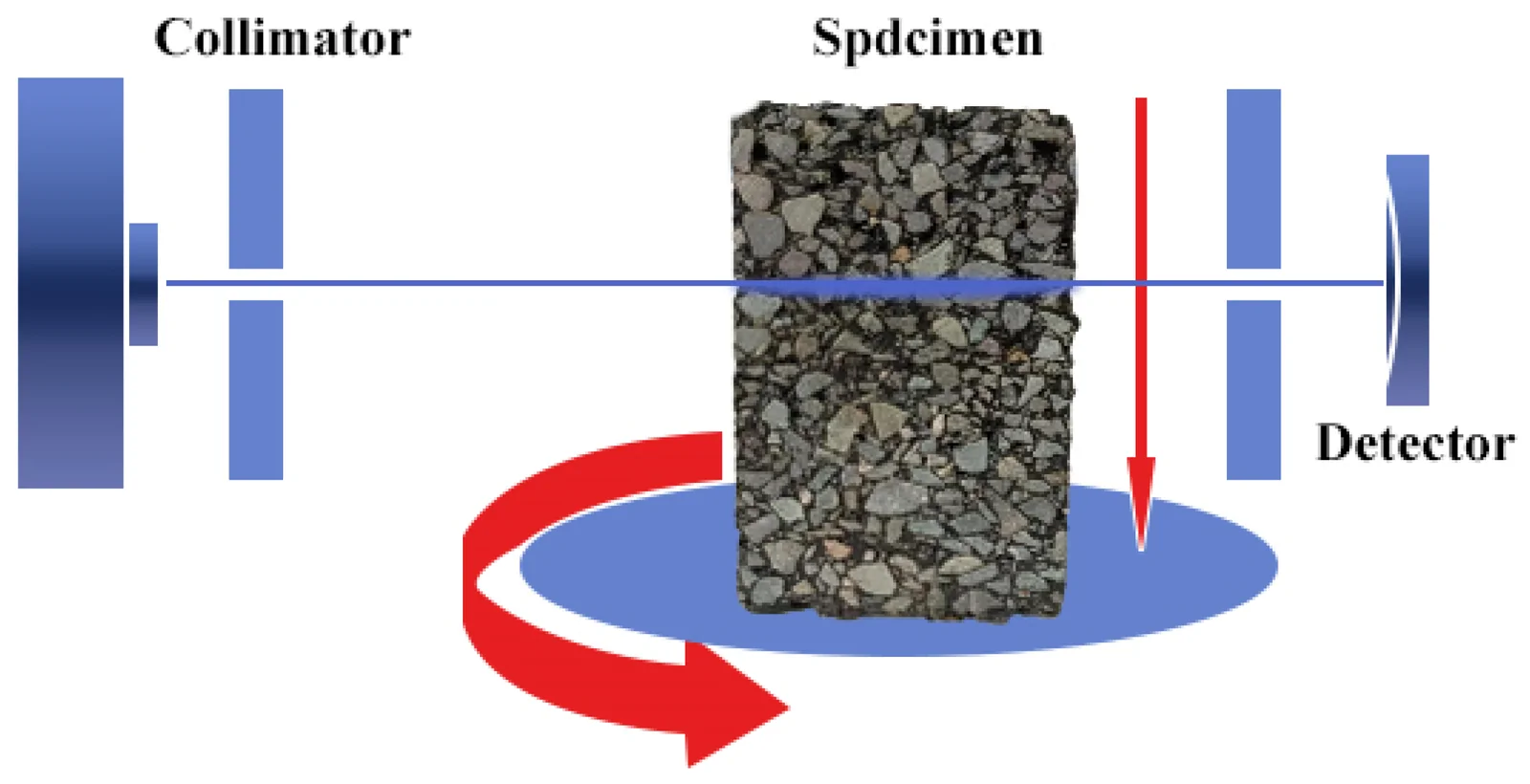
Schematic diagram of the CT scanning equipment.

Industrial X-ray CT was used to scan the specimens before and after immersion and freeze–thaw conditioning to characterize changes in their internal pore structure. Considering the self-healing characteristics of asphalt mixtures and the need to characterize sufficiently developed internal pore damage, CT scanning was performed on untreated specimens and specimens subjected to 12 days of immersion or 10 freeze–thaw cycles. A scanning interval of 0.13 mm was used, and the resulting CT images were subsequently processed to characterize the three-dimensional pore structure and the pore characteristics near the interlayer. A schematic diagram of the CT scanning equipment is shown in Figure 7.

## 3. Results and Analysis

### 3.1. Analysis of the Fatigue Performance

**Figure 8:**
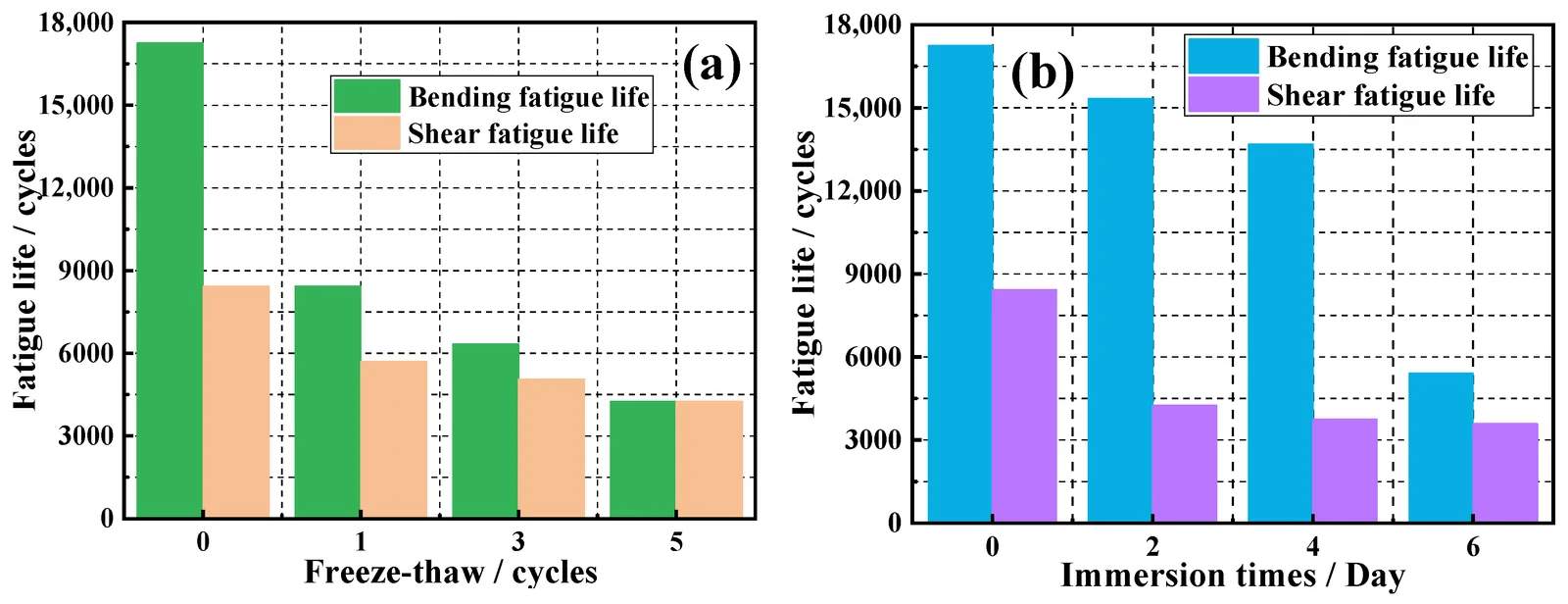
Fatigue life of specimens after freeze–thaw cycling and immersion: (**a**) freeze–thaw cycling, (**b**) immersion.

The fatigue life of the specimens after freeze–thaw cycling and immersion is shown in Figure 8.

It can be observed from Figure 8a that compared with the specimens without freeze–thaw cycles, after 1, 3, and 5 freeze–thaw cycles, the bending fatigue life of the specimens decreased by 51.1%, 64.3%, and 75.3%, respectively. The interlayer shear fatigue life decreased by 32.3%, 40.0%, and 49.6%, respectively. It can be concluded that both the bending fatigue performance and the interlayer shear fatigue performance of the specimens decreased after freeze–thaw cycling. Compared with the interlayer shear fatigue performance, the bending fatigue performance was more affected by freeze–thaw cycling.

It can be seen from Figure 8b that compared with the non-immersed specimens, the bending fatigue life of the specimens decreased by 11.1%, 20.6%, and 68.6%, respectively, after immersion for 2, 4, and 6 days. The interlayer shear fatigue life decreased by 49.6%, 51.6%, and 55.1%, respectively. Although the fatigue performance of the specimens after immersion showed a decreasing trend, the effects of immersion on the two fatigue properties were not exactly the same. The short-term immersion had no notable influence on the bending fatigue performance of the specimens. For the interlayer shear fatigue performance, the fatigue life decreased markedly after only 2 days of immersion. The results revealed that immersion had a pronounced effect on interlayer damage in the specimens.

### 3.2. Analysis of Mechanical Properties Within the ITZ

**Figure 9:**
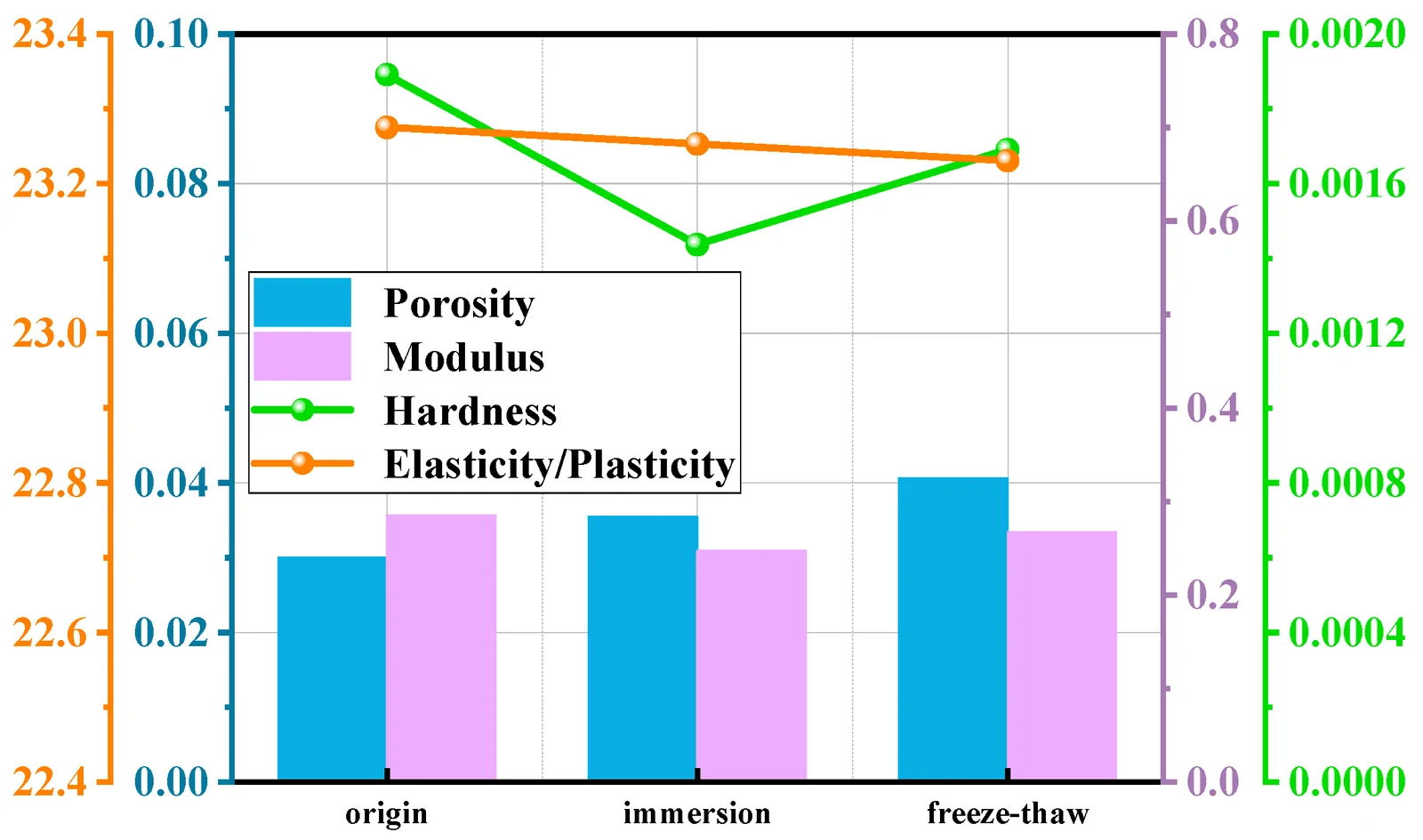
Micromechanical properties of the ITZ before and after immersion and freeze–thaw treatment.

The micromechanical properties of the asphalt mixture in the interfacial transition zone (ITZ) before and after immersion and freeze–thaw treatment were obtained by nanoindentation tests, as shown in Figure 9.

The results show that after exposure to water immersion and freeze–thaw cycles, the modulus of the ITZ in the specimens decreased to 0.2469 and 0.2672, respectively, representing reductions of 13.46% and 6.34% compared with the original specimen. Similarly, the hardness values after immersion and freeze–thaw cycles were measured at 0.00144 and 0.00169, showing decreases of 23.81% and 10.58% relative to the original specimen.

The original specimen had a modulus of 0.2853, which decreased to 0.2469 after water immersion. This indicates that water infiltration weakens the bond between asphalt and aggregate, leading to a reduction in the local stiffness of the ITZ. Due to pore expansion, the aggregate particles undergo displacement, resulting in a redistribution of the skeletal structure within the mixture. After freeze–thaw treatment, the modulus was 0.2672, which was 6.34% lower than that of the original specimen but higher than that of the immersed specimen. The difference between the two treated specimens may be related to the different effects of immersion and freeze–thaw cycling on the internal pore structure and aggregate arrangement.

The original hardness was 0.001892. After water immersion, it markedly decreased to 0.001437, indicating structural loosening and reduced resistance to localized penetration. After freeze–thaw treatment, the hardness was 0.001691, higher than that under the water-immersed condition but still lower than the original value. Therefore, both immersion and freeze–thaw treatment reduced the hardness of the ITZ.

Experimental results further show that the elastic/plastic ratios at the ITZ after water immersion and freeze–thaw cycles were 23.253 and 23.231, respectively, representing decreases of 0.10% and 0.19% compared with the original specimen. The ratio between elastic and plastic deformation decreased slightly after both treatments, and the values followed the order: original specimen > immersed specimen > freeze–thaw specimen.

### 3.3. Analysis of CT

#### 3.3.1. 3D Image Reconstruction

**Figure 10:**
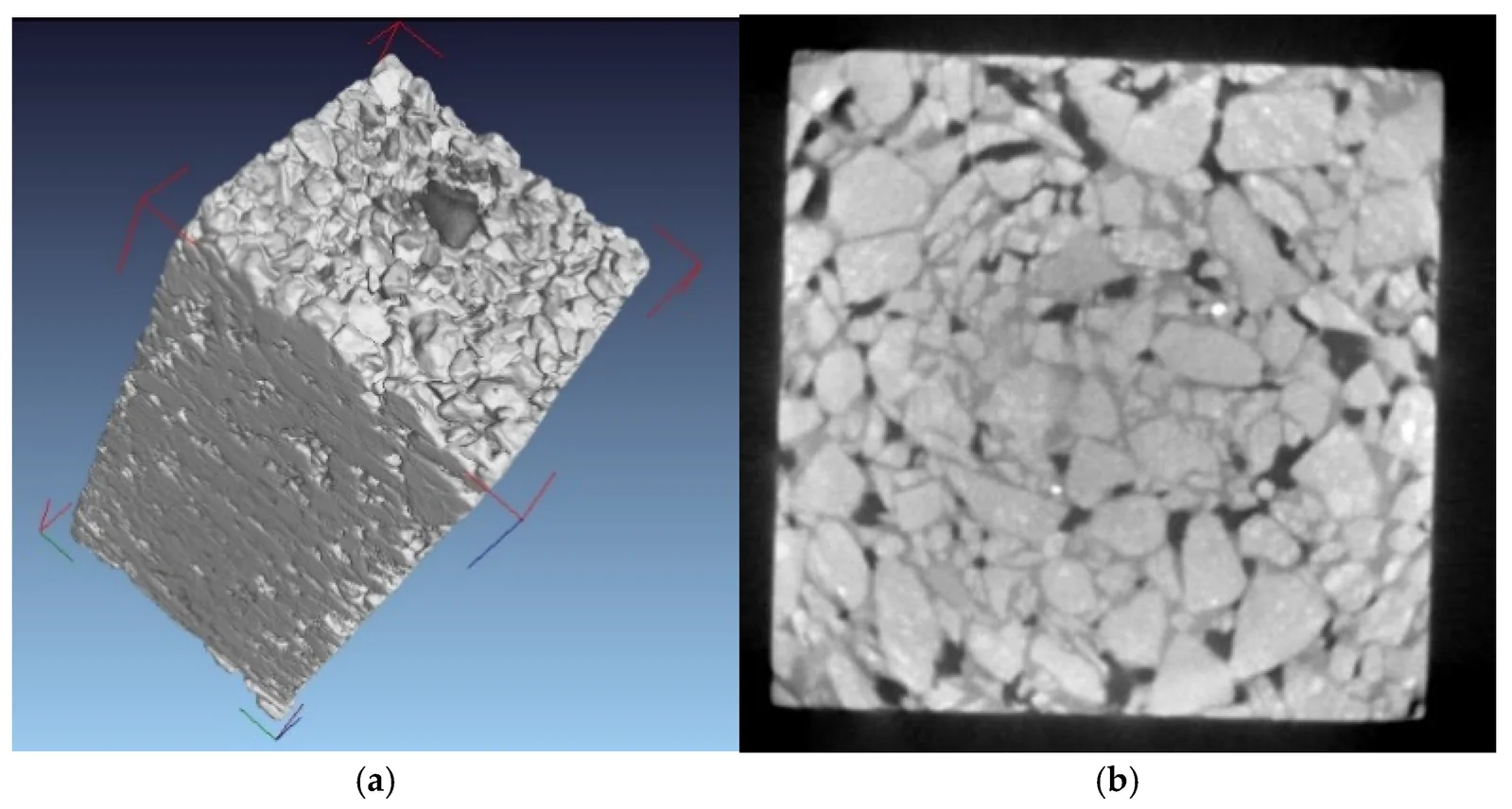
Schematic diagram of CT scanning: (**a**) scanning area, (**b**) two-dimensional slice image.

Because the specimen needed to be repositioned for each CT scan, to scan the overall morphological characteristics of the specimen, the scanning area was expanded, as shown in Figure 10a. Therefore, the obtained two-dimensional slice images contained black edge frames. Due to the imbalance of photons among voxels, the obtained images were affected by certain noise, as shown in Figure 10b.

**Figure 11:**
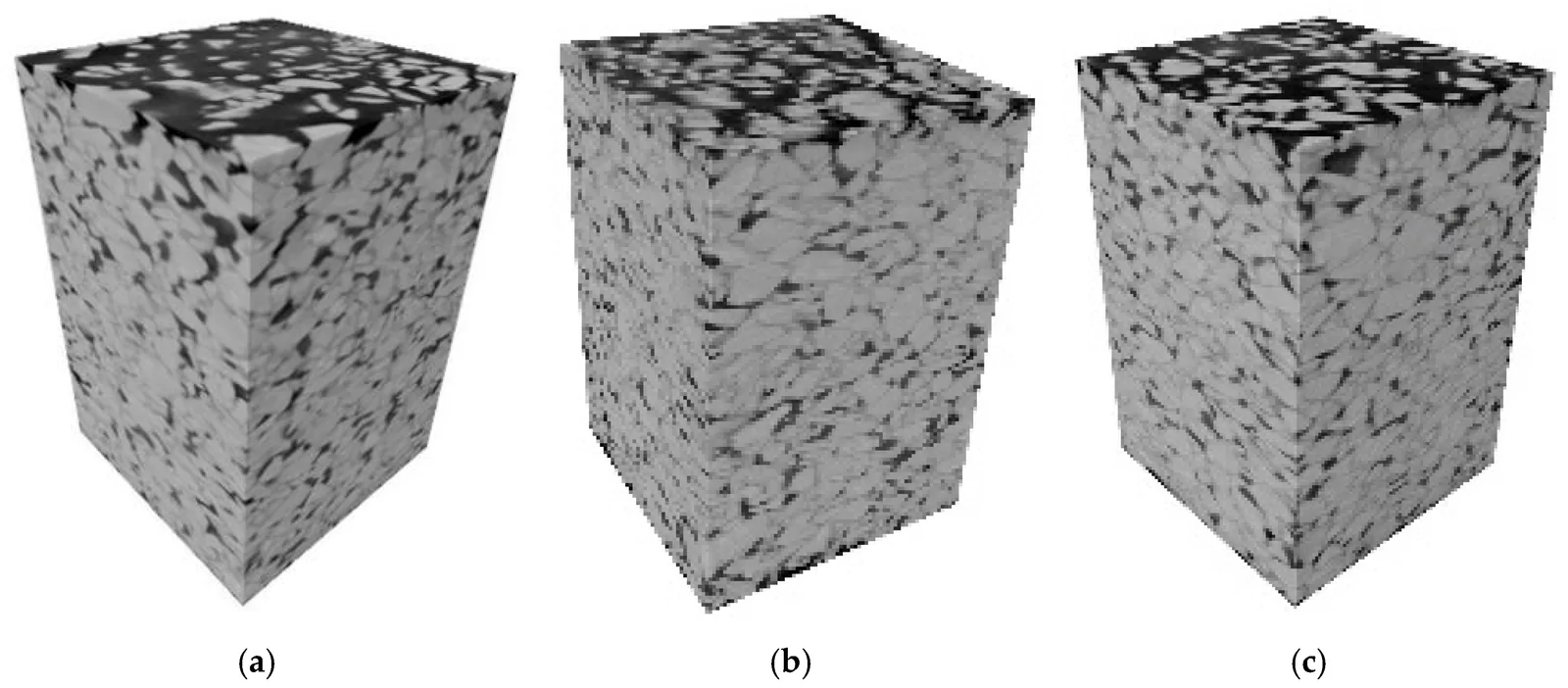
Three-dimensional structure diagram of the specimen: (**a**) original, (**b**) freeze–thaw, (**c**) immersion.

To solve the above problems, Avizo 9.5 software was used to process and analyze the two-dimensional images. When processing, the obtained two-dimensional images were first imported into the software, and then the median filter was used to denoise the images and reconstruct the three-dimensional structure. Because the specimen position varied slightly among scans, the reconstructed images could not completely overlap. Therefore, the Volume Edit module was used to crop the specimen to obtain a 50 mm × 50 mm × 90 mm specimen structure, as shown in Figure 11, in which black represents pores, gray represents asphalt mortar, and white represents aggregate.

#### 3.3.2. Pore Volume Distribution

**Figure 12:**
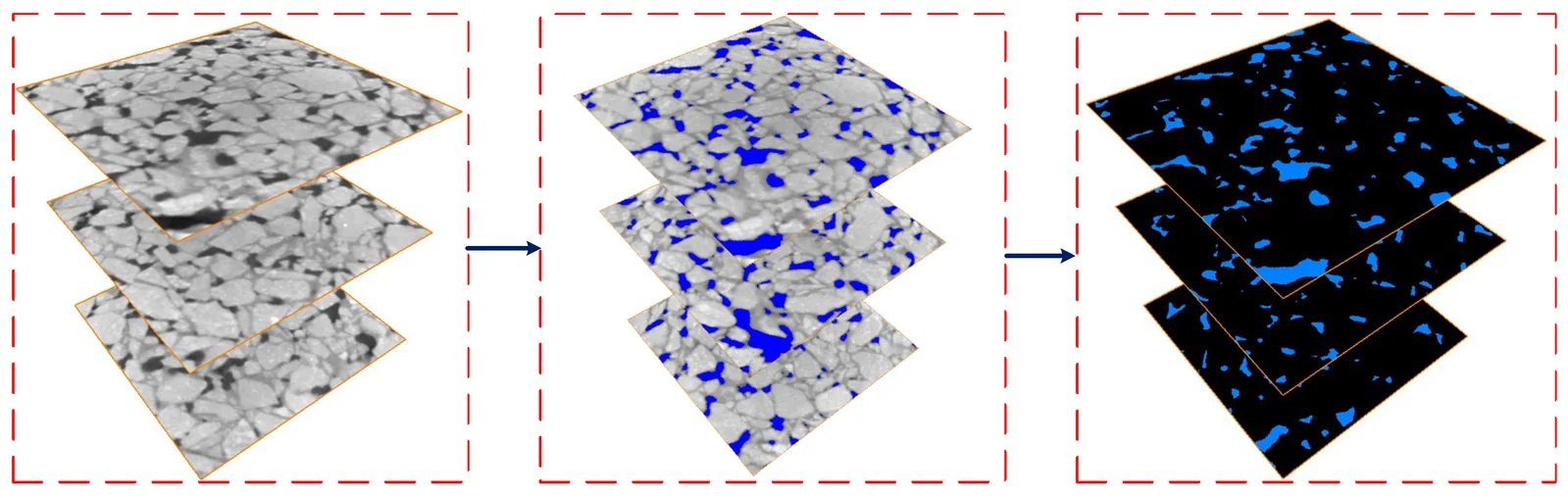
Image threshold segmentation process.

**Figure 13:**
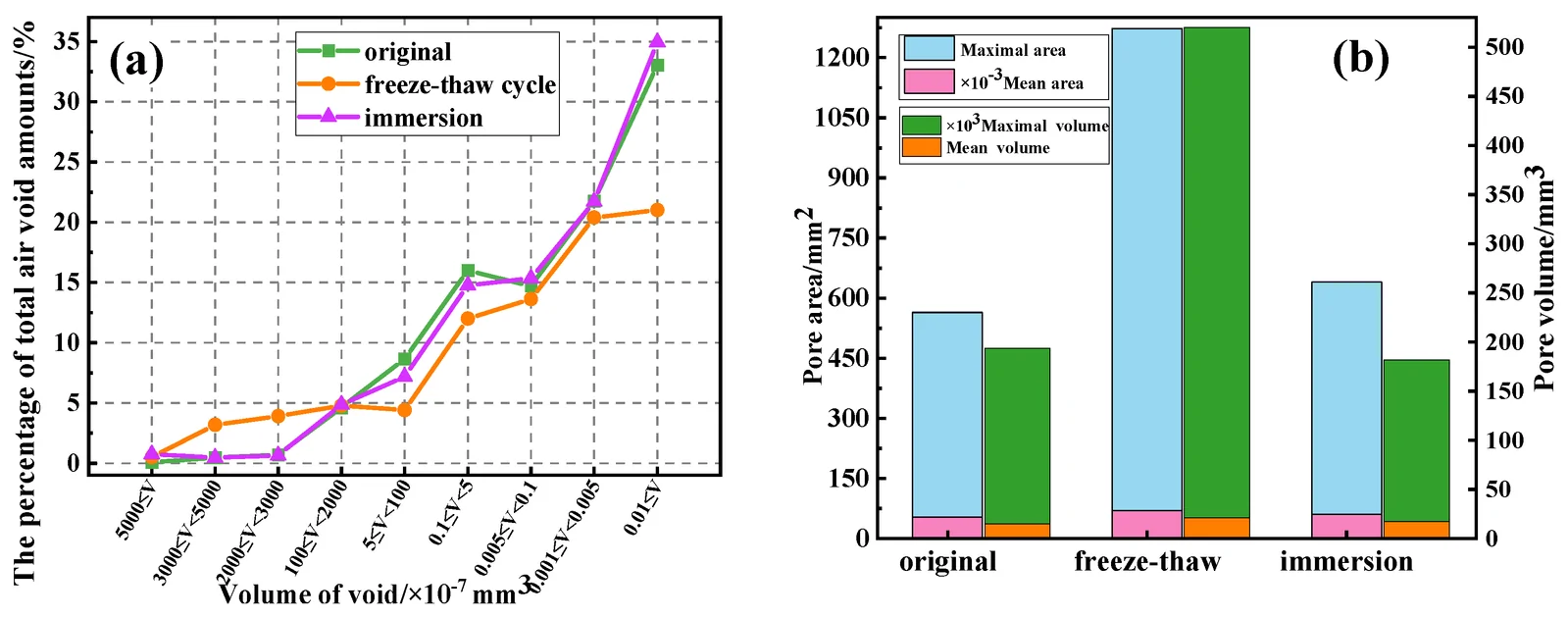
Distribution of pore characteristics: (**a**) pore volume distribution, (**b**) maximum and average values of pore area and volume.

To visually analyze the changes in the internal structure of the specimen, threshold segmentation was used to extract the pore structure within the specimen. Subsequently, the pores were reconstructed in 3D, and the volume of all pores within the specimen was calculated. The threshold segmentation process is illustrated in Figure 12. To evaluate the changes in pore-volume distribution within the specimen, the pores in the specimen were divided into nine ranges, and the number of pores within each range was counted. The variation in the number of pores within each range is shown in Figure 13a. The maximum and average values of the volume and area of the three specimens were calculated and presented in Figure 13b.

From Figure 13a, it can be observed that pores smaller than 0.1 mm^3^ occupy the largest proportion within the specimen. As the void volume increased, the proportion gradually decreased. Among the three specimens, there was not much difference in the proportion of pores with a volume larger than 20 mm^3^. By comparing the trends of the three curves, it can be concluded that the volume distribution of internal pores within the specimen did not change markedly before and after immersion. The proportion of pores with a volume of less than 0.1 mm^3^ increased by 5.6%, while the proportion of pores distributed within the range of 0.5–20 mm^3^ decreased by 4.2%. The distribution pattern of pore volumes was notably altered by freeze–thaw cycles, especially for pores with a volume larger than 20 mm^3^. After the freeze–thaw cycles, the proportion of these pores increased by 606.0%, while the proportion of pores with a volume less than 0.1 mm^3^ decreased by 36.4%. It can be observed that the internal pores within the specimen increased in size after the freeze–thaw cycles, resulting in a decrease in the bending fatigue performance of the specimen.

From Figure 13b, it can be observed that the average pore area and volume of the specimen increased after freeze–thaw and immersion treatments. Compared with the effect of immersion, the changes in the maximum area and volume of the specimen after freeze–thaw were considerable. The changes in area and volume after immersion were not remarkable, mainly because the additional pores were mostly smaller than 0.1 mm^3^, indicating that the immersion treatment did not markedly alter the volume and area distribution of pores within the specimen.

Based on the results of the freeze–thaw and immersion tests, it can be inferred from the relevant literature [41] that during the freeze–thaw cycling test, the volume of water in the pores gradually increased due to the phase change of water into ice, leading to an expansion of the original pore volume. Therefore, for asphalt mixtures, the volume of pores increased during the freeze–thaw cycles. During the immersion test, as the pH of the water used in the test was between 5 and 7, it was weakly acidic to nearly neutral. The hydrated hydrogen ions (H_3_O^+^) in the water may exchange with monovalent, divalent, or trivalent ions on the surface of basalt and generate activated complexes that promote its dissolution, such as (H_3_O) NaSiO_3_ and (H_3_O) MgSiO_6_. The reaction equations are shown in Equations (1) and (2). When these substances were dissolved or detached, the pores were filled with water, and when the specimen was completely dried, small pores could remain as a result of these water–rock reactions. Therefore, the internal structure of the mixture after immersion may undergo physicochemical changes associated with the water environment, promoting the formation of tiny pores inside the specimen.

(1) $$2H_{3}O^{+}+NaSiO_{3}^{-}\to H_{2}SiO_{3}+2H_{2}O+Na^{+}$$

(2) $$4H_{3}O^{+}+AlSi_{3}O_{8}^{-}\to H_{4}Si_{3}O_{8}+Al^{3+}+4H_{2}O$$

#### 3.3.3. Pore Size Distribution

**Figure 14:**
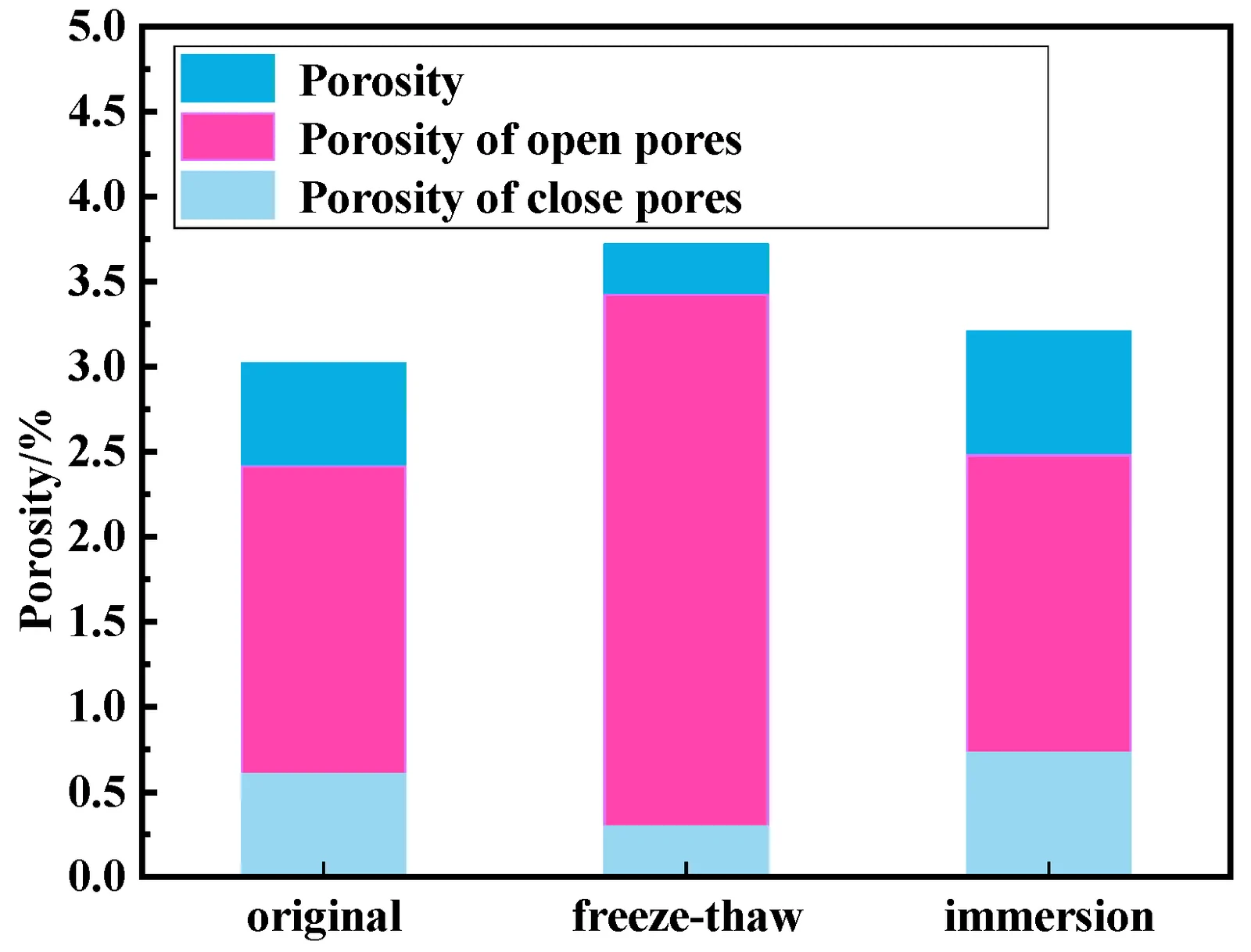
Proportion of open and closed pores.

To further study the distribution of internal pores in freeze–thaw and immersion specimens, open and closed pores, as well as connected and isolated pores within the specimens, were sequentially extracted. The proportions of open and closed pores in each specimen are shown in Figure 14.

From Figure 14, it can be observed that the porosity of the specimens increased by 23.88% and 6.32% after freeze–thaw and immersion treatments, respectively. After freeze–thaw, the proportion of closed pores in the specimens was only 7.95%, indicating that some closed pores gradually transformed into open pores due to pore expansion, making the specimens more prone to cracking and damage. In comparison with the original specimens, the increase in closed porosity was the main reason for the increase in total porosity after immersion, which was consistent with the previous conclusions.

**Figure 15:**
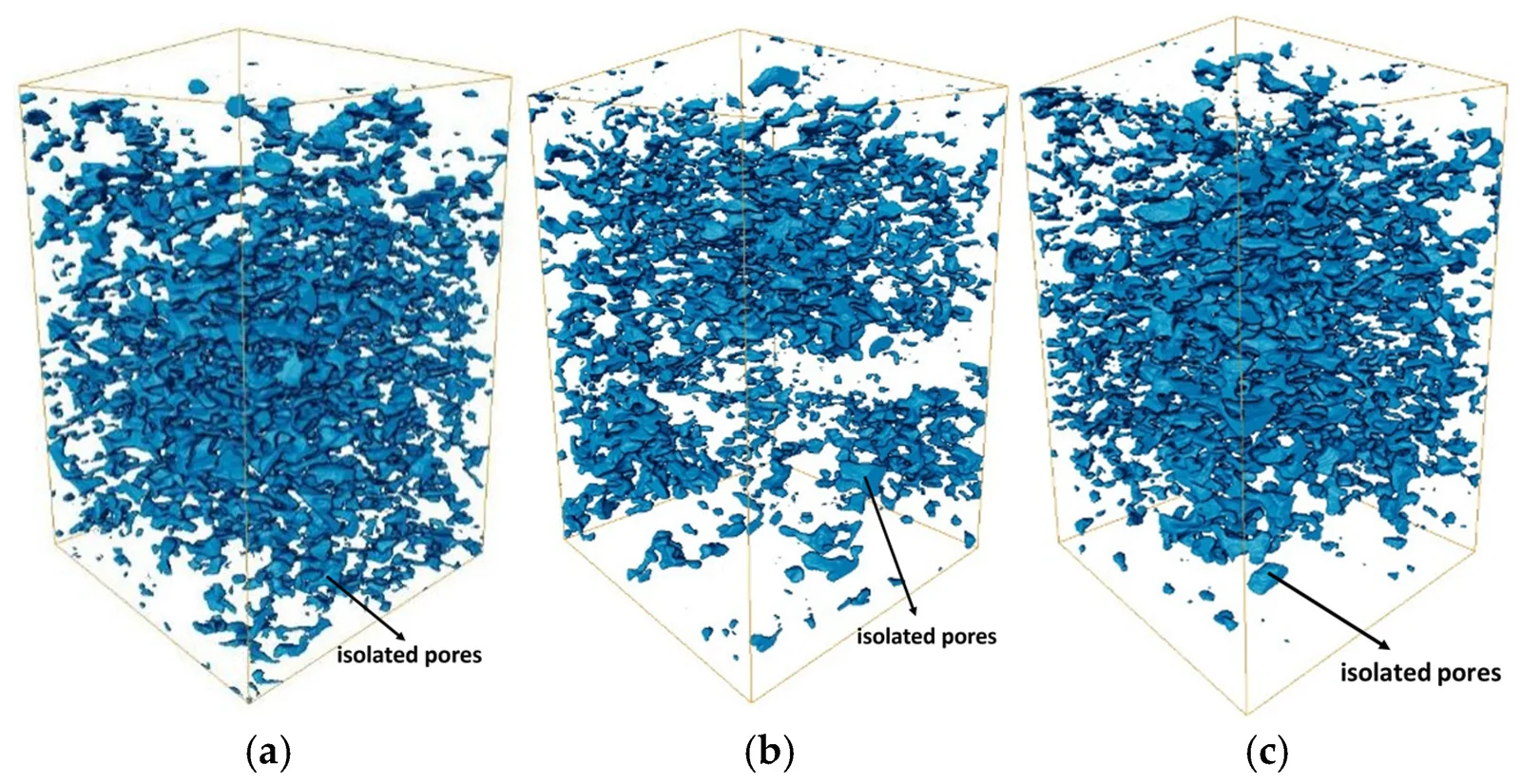
Isolated pore three-dimensional distribution diagram: (**a**) original, (**b**) freeze–thaw, (**c**) immersion.

**Figure 16:**
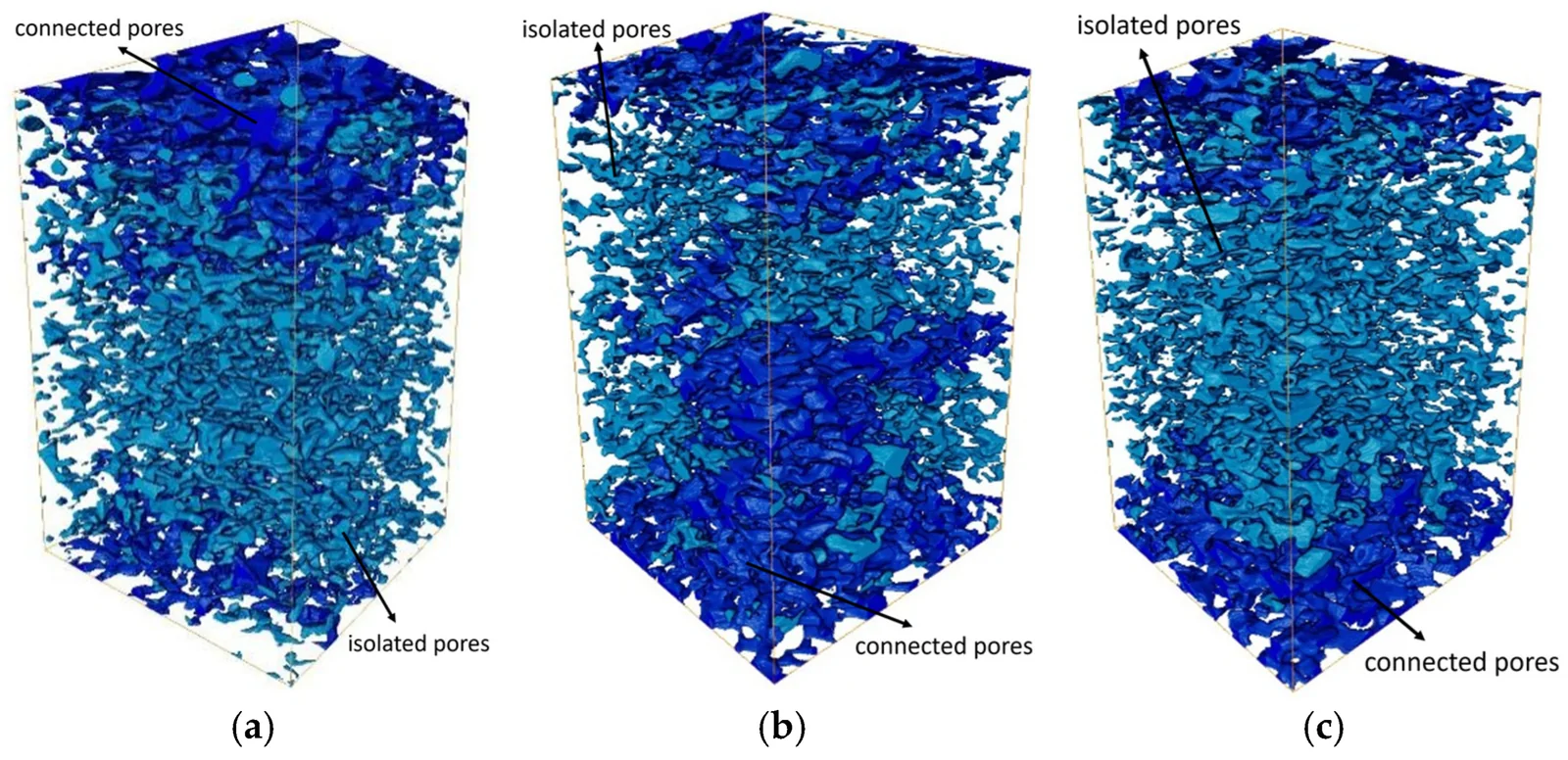
Connected-isolated pore three-dimensional distribution diagram: (**a**) original, (**b**) freeze–thaw, (**c**) immersion.

To analyze the distribution of connected and isolated pores within the freeze–thaw and immersion specimens, the Axis Connective module was used to extract the connected pores within the specimens, and then the And Not Image module was used to remove the connected pores from all pores, resulting in the isolated pores within the specimens, as shown in Figure 15. The three-dimensional spatial distribution of connected and isolated pores within the specimens is displayed in Figure 16. In Figure 15 and Figure 16, sky blue and dark blue represent isolated pores and connected pores, respectively.

From Figure 15 and Figure 16, it can be observed that the distribution of isolated pores in the asphalt mixture was relatively dense, mostly concentrated in the central region of the specimens, while connected pores were relatively dispersed and distributed mainly in the top and bottom regions of the specimens. Compared with the original specimens, the freeze–thaw treatment markedly reduced the number of isolated pores and increased the number of connected pores throughout the height of the specimens. After immersion, the number of isolated pores noticeably increased, especially near the interlayer, while the distribution of connected pores remained similar to that of the original specimens. It can be concluded that the increased number of connected pores in the freeze–thaw specimens was associated with the greater deterioration of the bending fatigue performance, while the increased number of isolated pores near the interlayer in the immersion specimens was associated with a marked reduction in interlayer shear fatigue life.

In summary, compared with immersion, freeze–thaw cycles caused more severe damage to the bending fatigue performance of asphalt mixtures. Therefore, in rainy and cold regions during winter, materials with denser structures should be used to reduce the influence of freeze–thaw on pore expansion, decrease the probability of isolated pores transforming into connected pores and closed pores transforming into open pores, thus avoiding the risk of crack propagation and material damage caused by open pores, and delaying the deterioration of road structural performance.

#### 3.3.4. Equivalent Pore Model

**Figure 17:**
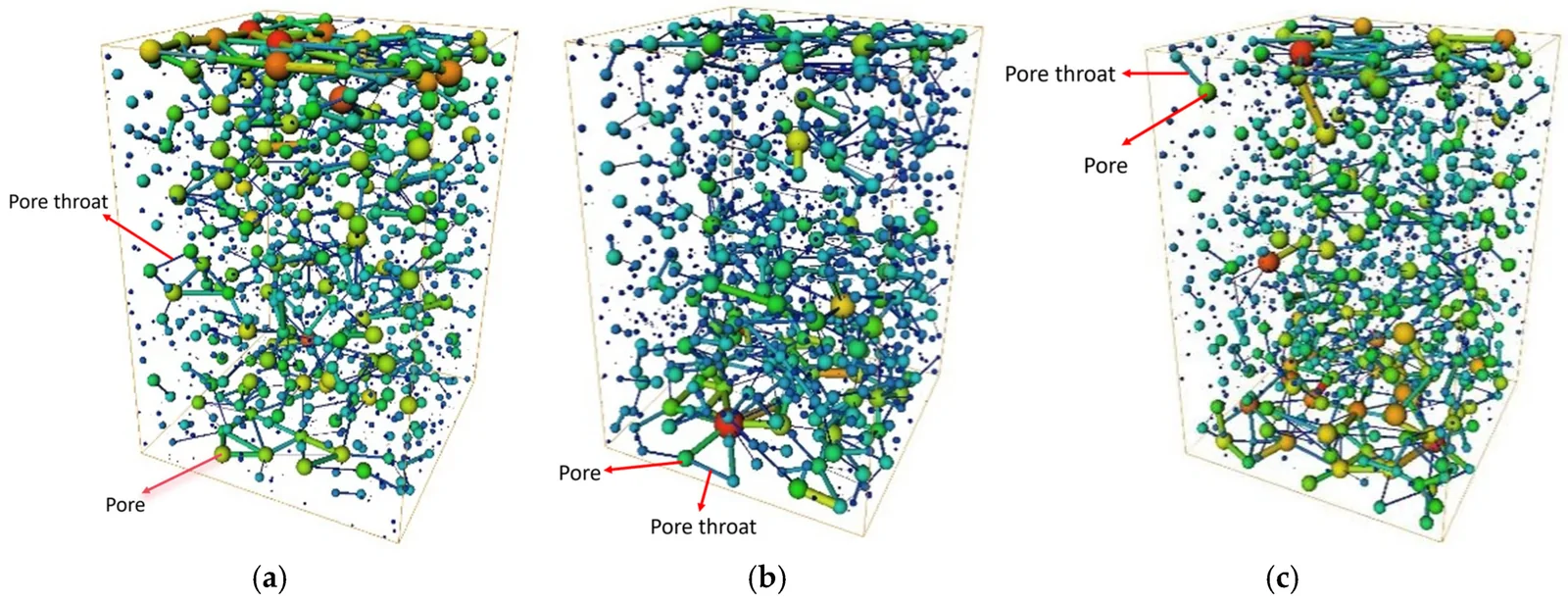
Equivalent pore structure diagram: (**a**) original, (**b**) freeze–thaw, (**c**) immersion.

To observe the distribution of pores more intuitively, the separate module was used to display the pore network structure in the material. Because the pores are not distributed in the specimen in a regular form, to uniformly characterize the size and position of the pores, the equivalent diameter of each pore was calculated. At the center of the pore shape, spherical particles were generated with the equivalent diameter as the diameter to characterize different pores, and bar-shaped structures were generated in the same way to characterize the pore throat connecting the pores, as illustrated in Figure 17.

It can be found from Figure 17 that compared with the original specimen, the pore throats of the specimen after freeze–thaw were mostly distributed in the lower part of the specimen, and the pore radius in the specimen increased together with the number of pore throats. After immersion, the pore throats of the specimen were also concentrated at the lower position of the specimen, and the pore distribution in the specimen was very dense. Therefore, the conclusion that the number of connected pores inside the specimen increased after freeze–thaw was inferred. Compared with the colors of the three figures in Figure 17, the pores in the original specimen were fewer and more uniformly distributed, and the amounts of pore throats and pores connected by pore throats were lower. After freeze–thaw cycling, the number of pore throats and connected pores increased. After immersion, the pore distribution became denser, although the total number of pore throats was slightly lower than that of the original specimen.

**Figure 18:**
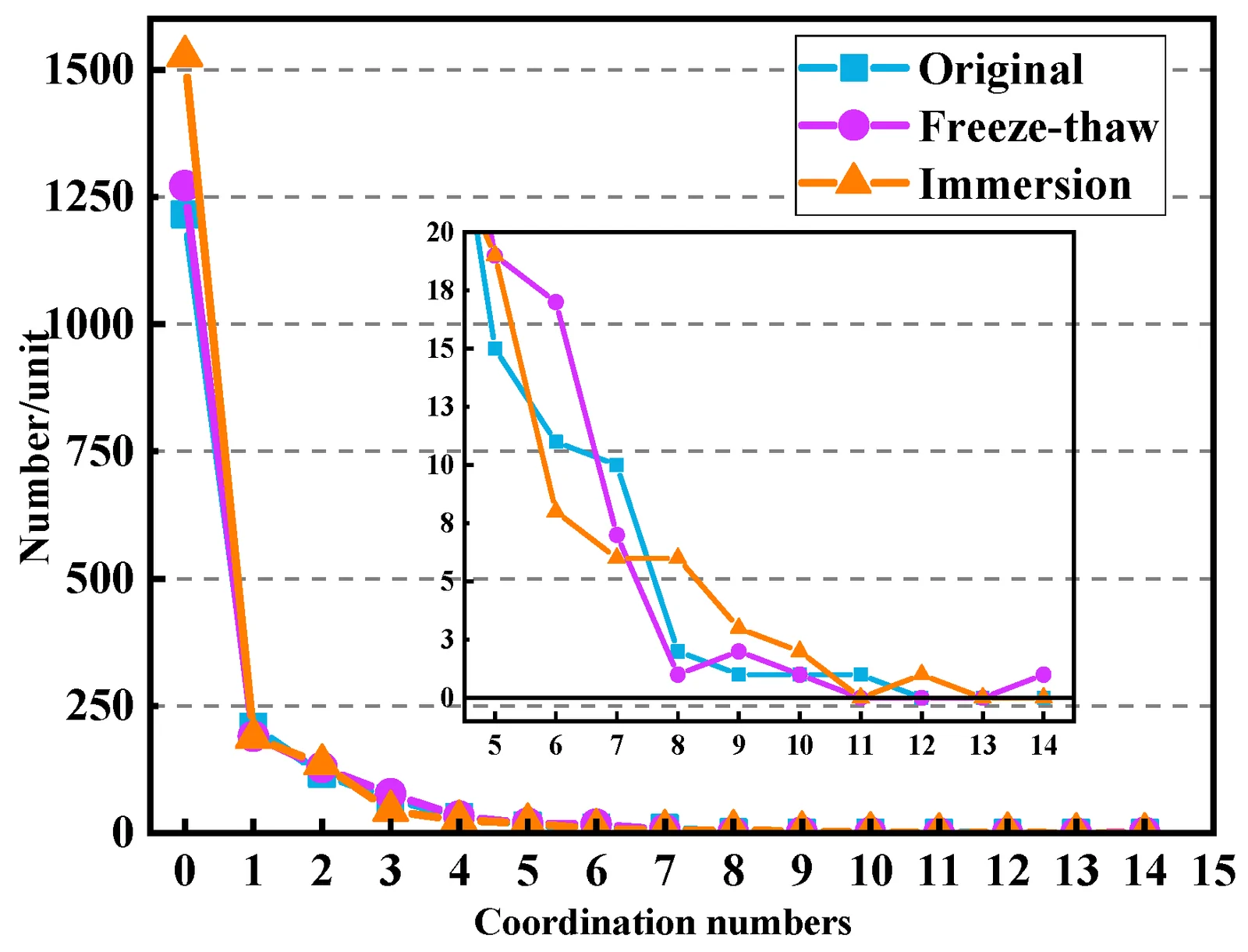
Variation trend of pore coordination number.

The coordination number refers to the number of pore throats connecting pores. To quantitatively analyze the changes in pores and pore throats in specimens, the coordination number of each pore was calculated, and the calculation results are shown in Figure 18.

Figure 18 shows that the coordination numbers of the three types of specimens showed a similar trend of first increasing and then decreasing. The maximum coordination numbers of the pores in the original specimen, freeze–thaw specimen and immersion specimen were 11, 14, and 12, respectively. Freeze–thaw treatment markedly increased the connectivity of the internal pores of the specimen. The proportion of pores with a coordination number of 0 in the immersed specimen increased by 20.48% compared with the original specimen, indicating that the isolated pores inside the specimen increased after immersion. The number of pores with a coordination number of 6 in the freeze–thaw specimen increased most markedly, indicating that the freeze–thaw treatment increased pore connectivity and promoted the formation of connected pores.

**Figure 19:**
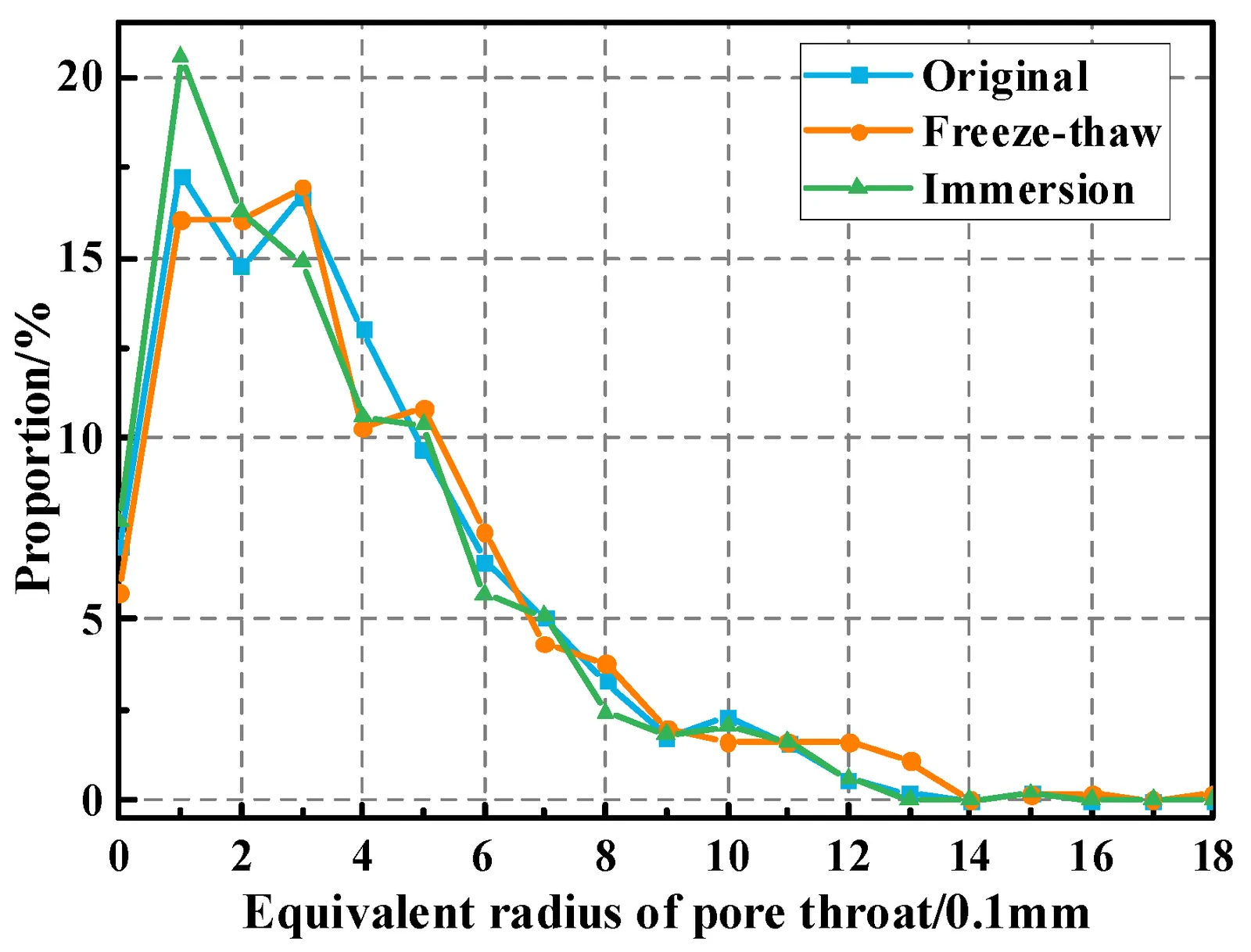
Distribution probability of pore throat radius.

Because the cross-sectional area of the pore throat is an important basis for evaluating the seepage performance of the specimen, the type of throat is not the key part of this study. Therefore, all the throats were regarded as bar-shaped in this paper, and the equivalent radius of the throat was used as an index to evaluate the cross-sectional area of each throat. The calculated pore-throat radii of the three specimens are shown in Figure 19.

It can be observed from Figure 19 that the number of throats of the three specimens increased first and then decreased with an increase in the equivalent radius of the pore throat, and the difference among the pore-throat-radius distributions was small. The peak values of the pore throats of the three specimens were distributed between 0.1~0.2 mm, 0.3~0.4 mm, and 0.1~0.2 mm, respectively. Although the proportion of pore throats distributed in the range of 0.1~0.2 mm after immersion was much higher than that of the original specimen, the connectivity of the specimen was not markedly affected due to the small radius of the pore throats.

The numbers of pore throats of the original specimen, the freeze–thaw specimen, and the immersion specimen were 515, 554, and 491, respectively. Pore throats can occur in different forms, including pore-shrinking, flake-like, tubular, and crack-like throats. Therefore, the reason for the decrease in the number of pore throats in the specimen after immersion may be due to the expansion of the original pore-shrinking throats to form pores, resulting in a decrease in the number of pore throats in the specimen. In contrast, freeze–thaw expanded the original pore volume and increased the proportion of open pores in the specimen, promoting the formation and expansion of cracks and increasing the number of pore throats in the specimen.

### 3.4. Pore Distribution at the Interlayer

**Figure 20:**
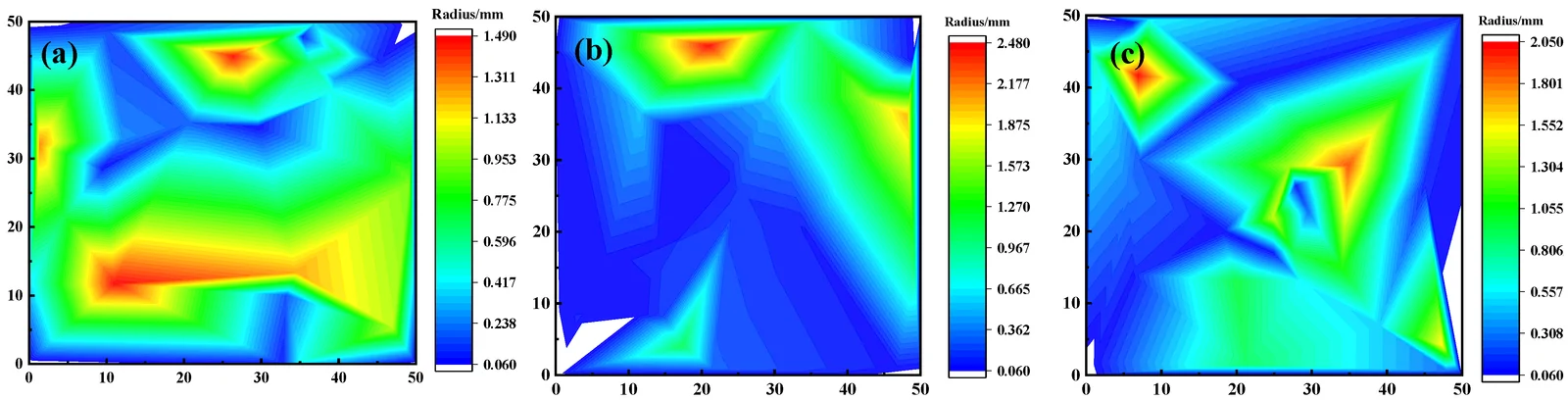
Pore-radius distribution at the interlayer: (**a**) original, (**b**) freeze–thaw, (**c**) immersion.

**Figure 21:**
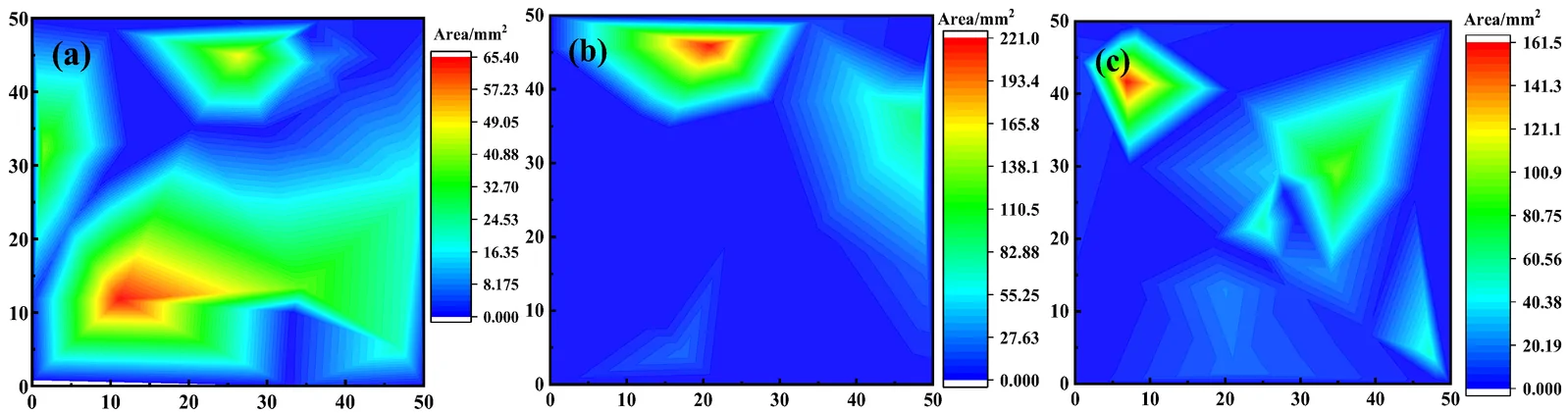
Pore-area distribution at the interlayer: (**a**) original, (**b**) freeze–thaw, (**c**) immersion.

**Figure 22:**
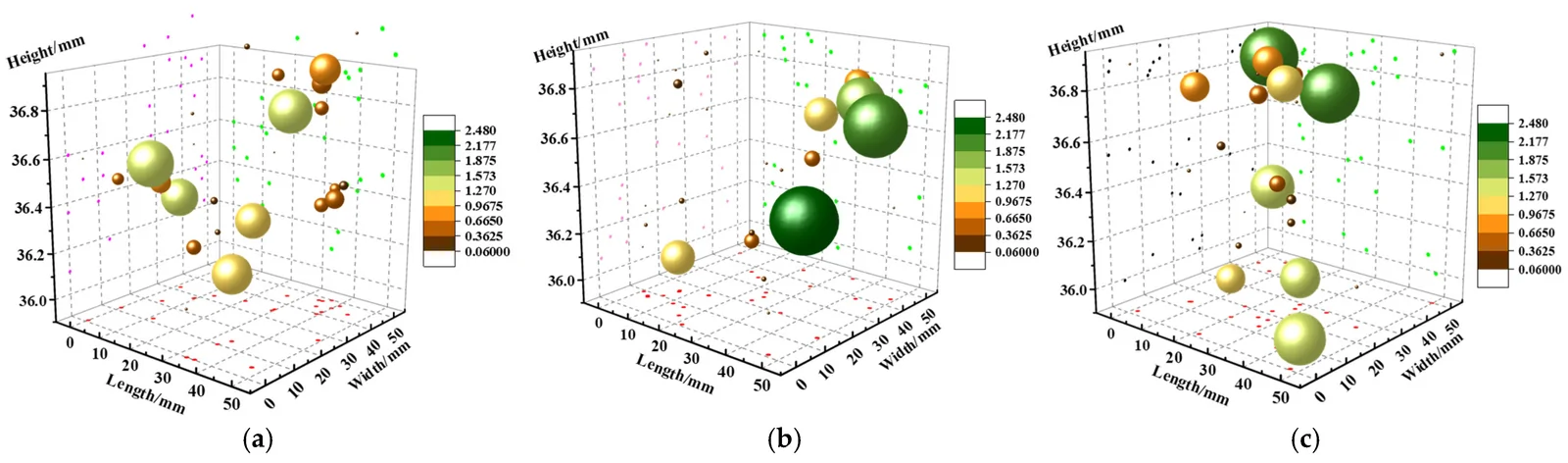
Pore-volume distribution at the interlayer: (**a**) original, (**b**) freeze–thaw, (**c**) immersion.

For the ultra-thin overlay, the bonding performance at the interlayer can directly affect the service life of the pavement. To analyze the changes in pore characteristics at the interlayer, the characteristic information of the pores within 0 ± 0.5 mm of the interlayer was extracted. The obtained pore distribution characteristics at the interlayer are shown in Figure 20 and Figure 21, and the volume distribution of the interlayer pores generated according to the equivalent radius of the pores is shown in Figure 22.

It can be observed from Figure 20 and Figure 21 that x and y are units of length and that the distribution of the equivalent radius and area of the same specimen at the interlayer position was basically the same, but because the pores were not regular spheres, the equivalent radius was not proportional to the area of the pores. It can be directly found from Figure 20 that the maximum pore areas of the original specimen, the freeze–thaw specimen and the immersion specimen were 65.4 mm^2^, 221.0 mm^2^, and 161.5 mm^2^, respectively. It can be concluded that the maximum radius of the pores at the interlayer after freeze–thaw was about 2.0 times that of the original specimen, and the maximum pore area was about 3.4 times that of the original specimen. The maximum radius of the pores at the interlayer after immersion was about 1.4 times that of the original specimen, and the maximum pore area was about 2.5 times that of the original specimen.

By observing the distribution of pore volume at the interlayer in Figure 22, it can be found that although the maximum area and maximum radius of the pores after immersion were smaller than those of the specimens after freeze–thaw, the pore distribution at the interlayer after immersion was denser, which was consistent with the results of the dense distribution of isolated pores at the interlayer in Section 3.3.3. Therefore, the interlayer shear fatigue performance of the specimens after immersion was slightly lower than that after freeze–thaw cycles.

Through calculation, the numbers of pore throats at the interlayer in the original specimen, freeze–thaw specimen, and immersion specimen were 0, 13, and 4, respectively. Although the number of pore throats at the interlayer increased markedly after freeze–thaw, the relatively small pore-throat radius indicates that the change in the number of pore throats alone was not the key factor affecting the interlayer shear fatigue performance.

### 3.5. Pore Tortuosity

Changes in the internal pore structure can cause material damage, reduce mechanical performance, and eventually lead to fatigue, fracture, and failure. Tortuosity is a parameter used to characterize the complexity and continuity of pore pathways in materials. To study the influencing factors of pore tortuosity in asphalt mixtures, the adaptive-network-based fuzzy inference system (ANFIS) was used to predict pore tortuosity based on pore characteristic parameters. The equivalent diameter, volume shape factor, and porosity were used as input variables, and tortuosity was the output variable. The model combines an adaptive neural network and a fuzzy inference system. It retains the interpretability of the fuzzy inference system and the learning ability of the adaptive neural network, allowing the predicted values to approach the measured values through iterative learning.

Firstly, the input values of *x* and *y* were fuzzified by membership functions, and a membership degree *μ* was obtained [42,43], as shown in Equation (3). A value of 0 indicates that the element *x* is not a fuzzy set element, a value of 1 indicates that the element *x* is a fuzzy set element, and a value between 0 and 1 indicates that this element partially conforms to the fuzzy set.

(3) $$O_{i}^{1}=\mu_{A_{i}}(x),\;i=1,\;2\;\mathrm{or}\;O_{i}^{1}=\mu_{B_{i}}(y),\;i=1,\;2$$

where *x* and *y* are the input values of node *i*, *A_i_* and *B_i_* are fuzzy sets; $O_{i}^{1}$ is the membership function value of *A_i_* and *B_i_*, indicating the degree of *x* and *y* belonging to *A_i_* and *B_i_*; the shape of the membership functions $\mu_{A_{i}}$, $\mu_{B_{i}}$ is determined by the antecedent parameters. Usually, the function with a value between 0 and 1 is selected, for example,

(4) $$\mu_{Ai}(x)=e^{-{\left(\frac{x-c_{i}}{a_{i}}\right)}^{2}}$$

where *c_i_* is the center of a Gaussian function; *a_i_* is the width of the Gaussian function; *a_i_* and *c_i_* is a parameter that needs to be optimized.

Secondly, the membership degree of each variable was multiplied, as shown in Equation (5), and the firing strength of each rule was obtained. This layer is called the strength release layer of the rule, and the output of each node represents the credibility of the rule.

(5) $$O_{i}^{2}=\omega_{i}=\mu_{A_{i}}(x)\times \mu_{B_{i}}(y)$$

where *ω_i_* is the output of the second layer.

Then, the firing strength of each rule in the second layer was normalized, as shown in Equation (6), which represents the trigger proportion of the rule in the whole rule base.

(6) $$O_{i}^{3}=\bar{\omega_{i}}=\frac{\omega_{1}}{\omega_{1}+\omega_{2}},\;i=1,\;2$$

where $\bar{\omega_{i}}$ is the output of the third layer.

In the fourth layer, the variable *x* needs to be input again. Each node *i* in this layer is an adaptive node, and its output is

(7) $$O_{i}^{4}=\bar{\omega_{i}}f_{i}=\bar{\omega_{i}}(p_{i}x+q_{i}y+r_{i}),\;i=1,\;2$$

where $\bar{\omega_{i}}$ is the output of the third layer and $\left\{p_{i}\right.,q_{i},\left.r_{i}\right\}$ is the parameter set of the node, which is called the consequent parameter.

Finally, the defuzzification was performed to obtain the exact result, as shown in Equation (8), which was the weighted average of each rule result output by the system.

(8) $$O_{i}^{5}=\sum \bar{\omega_{i}}f_{i}=\frac{\sum \omega_{i}f_{i}}{\sum \omega_{i}},\;i=1,\;2$$

When the antecedent parameters are given, the output value of ANFIS can be expressed as follows:

(9) $$O_{i}^{5}=\bar{\omega_{1}}f_{1}+\bar{\omega_{2}}f_{2}=(\bar{\omega_{1}}x)p_{1}+(\bar{\omega_{1}}y)q_{1}+(\bar{\omega_{1}})r_{1}+(\bar{\omega_{2}}x)p_{2}+(\bar{\omega_{2}}y)q_{2}+(\bar{\omega_{2}})r_{2}$$

The learning algorithm adopted by ANFIS in this paper is the LSE-GD hybrid algorithm, which updates parameters through forward and backward passes. In the forward learning process of the network, *n* sets of training data are used, and the premise parameters are fixed. When the calculation reaches the fourth layer, the consequent parameters are determined using the least-squares estimation (LSE) method, and the error between the predicted and target values is propagated backward to update the premise parameters using the gradient descent (GD) method.

In reverse learning, the parameters in each rule are specified, and the partial derivatives of the measurement error for each parameter are obtained by using the chain rule according to the LOSS function, as shown in Equations (10) and (11); this enables us to obtain the partial derivatives of the antecedent parameters and update the parameters from the opposite direction of the gradient direction. The error function was calculated from the difference between the target output and the actual output, as shown in Equation (12).

(10) $$\frac{\partial E_{p}}{\partial O_{i,p}^{L}}=-2(T_{i,p}-O_{i,p}^{L})$$

(11) $$\frac{\partial E_{p}}{\partial \alpha }=\sum_{O^{*}\varepsilon S}\frac{\partial E_{p}}{O^{*}}\frac{\partial O^{*}}{\partial \alpha }$$

(12) $$E_{p}=\sum_{m=1}^{\mu (L)}{\left(T_{m,p}-O_{m,p}^{L}\right)}^{2}$$

where *T_m,p_* is the *m*-th component of the *p*-th target output; $O_{m,p}^{L}$ is the m-th component of the *L*-th layer of the *p*-th actual output; E represents the root-mean-square error in one training, and its value is equal to the sum of the mean square errors of all *P* outputs.

**Figure 23:**
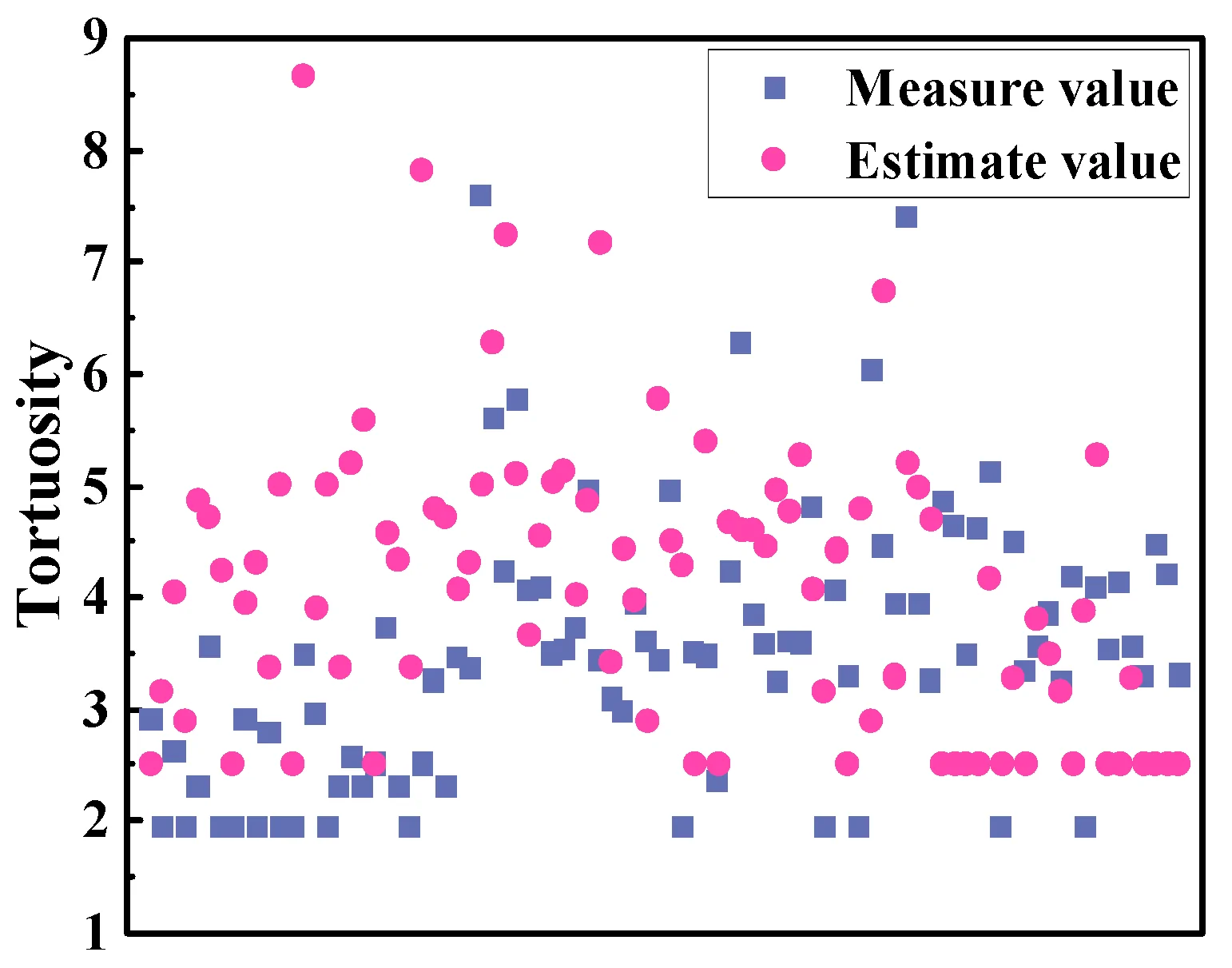
The calculated value and the predicted value of tortuosity.

The calculated and predicted values of the pore tortuosity of the original specimen obtained by training are shown in Figure 23. The abscissa in Figure 23 represents the pore index assigned when the training dataset was input. For the predicted model, the training data errors of the original, freeze–thaw, and immersed specimens were 0.119, 0.102, and 0.134, respectively, and the test data errors were 0.129, 0.102, and 0.115, respectively. The calculated and predicted values showed good agreement, indicating that the model can be used to predict pore tortuosity.

The prediction results indicate that equivalent diameter, shape factor, and porosity are closely related to pore tortuosity. Some scholars have proposed that the porosity, pore size, and shape factor of the pores can be combined into the pore comprehensive effect index (PCEI) [44], as shown in Equation (13). To analyze the functional relationship between tortuosity and PCEI, the PCEI of each specimen was calculated, as shown in Figure 22.

(13) $$\gamma_{PCEI}=\frac{R_{p}}{{\left(1-\varphi \right)}^{\frac{1}{SF}}}$$

where *R_p_* is the pore radius, mm; *φ* is the porosity of the specimen; and *SF* is the shape factor of the pore.

**Figure 24:**
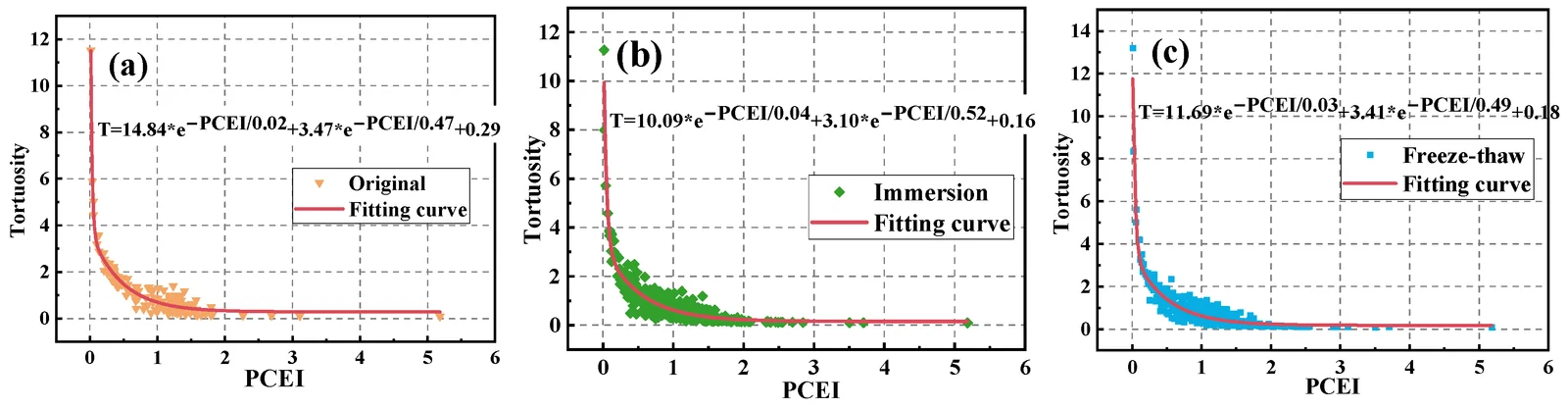
Relations between PCEI and tortuosity: (**a**) original, (**b**) freeze–thaw, (**c**) immersion.

It can be observed from Figure 24 that by fitting the scatter points in the figure, an exponential relationship was obtained between pore tortuosity and PCEI. The pore tortuosity gradually decreased with an increase in shape factor *SF* and porosity, and gradually increased with an increase in pore radius. Therefore, the tortuosity of the pores inside the specimen can be calculated, and then the characteristics of the pores can be analyzed to evaluate the performance of the asphalt mixture.

### 3.6. Discussion

Freeze–thaw cycling and water immersion produced different deterioration patterns in the SBS/MCF composite-modified asphalt mixture. As the number of freeze–thaw cycles increased, both bending fatigue life and interlayer shear fatigue life decreased, with a larger reduction observed in bending fatigue life. Similar fatigue deterioration under repeated freeze–thaw action has been reported in previous studies [36,45]. This behavior is likely related to the progressive development of internal damage during freezing and thawing. Expansion and connection of pores can promote microcrack propagation and gradually weaken the load-bearing skeleton, making the overall bending response particularly sensitive to freeze–thaw damage.

The effect of immersion was different. Interlayer shear fatigue life decreased markedly during the early stage of immersion, whereas bending fatigue life changed only slightly after short-term immersion. Previous studies have also reported that moisture can significantly weaken interlayer bonding in asphalt pavement structures [27,32]. In the present study, this response may be associated with water entering the interlayer region and reducing the bonding between the tack coat, asphalt mastic, and aggregate surfaces. As a result, interlayer deterioration can occur before substantial damage develops throughout the whole specimen, which explains the greater sensitivity of shear fatigue performance to short-term immersion.

The CT results are consistent with these different deterioration mechanisms. Earlier studies have shown that freeze–thaw cycling can enlarge existing pores, connect previously separated pores, and generate new pores [36,46]. In this study, total porosity increased by 23.88% after freeze–thaw treatment, together with a clear increase in connected pores. By comparison, immersion increased total porosity by only 6.32%, while the increase was mainly associated with small and isolated pores, particularly near the interlayer. These differences suggest that freeze–thaw damage is more closely related to pore enlargement and connectivity, whereas immersion mainly causes localized deterioration near the interface. Accordingly, porosity alone is not sufficient to explain the changes in fatigue performance; pore type, connectivity, and spatial distribution also need to be considered.

The nanoindentation results provide further support for this interpretation. The ITZ modulus and hardness after immersion were lower than those after freeze–thaw treatment, although freeze–thaw cycling caused a greater reduction in bending fatigue life. The difference is reasonable because the two tests reflect damage at different scales. Nanoindentation characterizes the local mechanical response of the asphalt–aggregate interface, while bending fatigue depends more strongly on the integrity of the overall internal skeleton and the development of damage through the specimen. Thus, immersion produced more pronounced local weakening at the ITZ, whereas freeze–thaw cycling caused more extensive pore-network deterioration. The relationship between PCEI and tortuosity obtained in this study also indicates that pore geometry and connectivity should be considered together when evaluating environmental deterioration.

## 4. Conclusions

Through three-point bending fatigue, direct shear fatigue, nanoindentation, and CT scanning tests on interlayer asphalt mixture specimens subjected to freeze–thaw cycling and immersion, the main conclusions are as follows:

(1) Compared with untreated specimens, freeze–thaw cycling caused a continuous decrease in both bending and interlayer shear fatigue life. After five freeze–thaw cycles, the bending fatigue life decreased by 75.3%, while the interlayer shear fatigue life decreased by 49.6%. Under immersion, the bending fatigue life decreased slowly at the initial stage and markedly after prolonged immersion, whereas the interlayer shear fatigue life decreased rapidly at the initial stage and decreased by 55.1% after six days of immersion. These results show that freeze–thaw cycling and immersion had different effects on the fatigue performance of the interlayer asphalt mixture.
(2) Freeze–thaw action induces pore expansion and aggregate displacement, leading to the restructuring of the asphalt mixture’s skeleton. Both the modulus and hardness of the ITZ after freeze–thaw exposure were slightly higher than those after water immersion. However, under both conditions, moisture intrusion weakened the asphalt–aggregate interface, resulting in an overall reduction in stiffness and hardness. The ratio characterizing elastic and plastic deformation also decreased slightly after both treatments in the following order: original specimen > immersed specimen > freeze–thaw specimen. The nanoindentation results show that the mechanical properties of the ITZ deteriorated after both environmental treatments.
(3) Freeze–thaw treatment mainly promoted pore enlargement and increased pore connectivity, whereas immersion mainly increased the number of small pores and isolated pores. After freeze–thaw treatment, part of the closed pores transformed into open pores, and the number of connected pores increased. After immersion, the proportion of pores with a coordination number of 0 increased by approximately 20%, and isolated pores were mainly concentrated near the interlayer. The changes in fatigue performance were therefore related not only to porosity, but also to pore type, connectivity, and spatial distribution.
(4) Both freeze–thaw cycling and immersion changed the pore characteristics near the interlayer. The maximum pore area at the interlayer increased from 65.4 mm^2^ in the original specimen to 221.0 mm^2^ and 161.5 mm^2^ after freeze–thaw and immersion treatments, respectively. Freeze–thaw treatment increased the number of interlayer pore throats, whereas immersion resulted in a denser pore distribution near the interlayer. The number and distribution of pores near the interlayer were more closely related to interlayer shear fatigue performance than the number of pore throats alone.
(5) ANFIS analysis showed that equivalent diameter, shape factor, and porosity were related to pore tortuosity. The testing errors of the untreated, freeze–thaw, and immersed specimens ranged from 0.102 to 0.129. An exponential relationship was obtained between PCEI and tortuosity, providing a quantitative approach for further characterizing pore connectivity. These results provide a basis for evaluating the fatigue deterioration and pore-structure evolution of SBS/MCF composite-modified asphalt mixtures used in ultra-thin overlays under freeze–thaw and immersion conditions.

## Figures and Tables

**Table 1:** Technical properties of SBS/MCF composite-modified asphalt.

Property	Unit	Value
Penetration (25 °C, 100 g, 5 s)	0.1 mm	53
Penetration Index (PI)	-	0.2
Ductility (5 °C, 5 cm/min)	cm	31
Softening Point (R&B)	°C	79.5
Flash Point	°C	298
Rotational Viscosity (135 °C)	Pa·s	1.6
Solubility	%	99.95
Elastic Recovery (25 °C)	%	90
Difference in Softening Point after TFOT	°C	1.9
Density (25 °C)	g/cm^3^	1.023

## Data Availability

The original contributions presented in this study are included in the article. Further inquiries can be directed to the corresponding author.

## Abbreviations

The following abbreviations are used in this manuscript:

SBS	Styrene–butadiene–styrene
MCF	Micro carbon fiber
